# MAP6 interacts with Tctex1 and Ca_v_2.2/N‐type calcium channels to regulate calcium signalling in neurons

**DOI:** 10.1111/ejn.13766

**Published:** 2017-11-22

**Authors:** Jacques Brocard, Fabrice Dufour, Sylvie Gory‐Fauré, Christophe Arnoult, Christophe Bosc, Eric Denarier, Leticia Peris, Yasmina Saoudi, Michel De Waard, Annie Andrieux

**Affiliations:** ^1^ U1216 INSERM Grenoble F‐38000 France; ^2^ Grenoble Institute of Neuroscience Université Grenoble Alpes Grenoble France; ^3^ U1209 INSERM Grenoble France; ^4^ UMR 5309 CNRS Grenoble France; ^5^ Institute for Advanced Biosciences Université Grenoble Alpes Grenoble France; ^6^ CEA, BIG‐GPC Grenoble France; ^7^ U1087 INSERM Nantes France; ^8^ UMR 6291 CNRS Nantes France; ^9^ Université Nantes Nantes France

**Keywords:** hippocampal neurons, *in vitro* interactions, microtubules, plasma membrane, vesicles, yeast two‐hybrid

## Abstract

MAP6 proteins were first described as microtubule‐stabilizing agents, whose properties were thought to be essential for neuronal development and maintenance of complex neuronal networks. However, deletion of all MAP6 isoforms in MAP6 KO mice does not lead to dramatic morphological aberrations of the brain but rather to alterations in multiple neurotransmissions and severe behavioural impairments. A search for protein partners of MAP6 proteins identified Tctex1 – a dynein light chain with multiple non‐microtubule‐related functions. The involvement of Tctex1 in calcium signalling led to investigate it in MAP6 KO neurons. In this study, we show that functional Ca_v_2.2/N‐type calcium channels are deficient in MAP6 KO neurons, due to improper location. We also show that MAP6 proteins interact directly with both Tctex1 and the C‐terminus of Ca_v_2.2/N‐type calcium channels. A balance of these two interactions seems to be crucial for MAP6 to modulate calcium signalling in neurons.

## Introduction

MAP6 proteins were first described as microtubule‐associated agents protecting microtubules vis‐à‐vis cold temperatures [coined STOP for Stable Tubule Only Polypeptides (Margolis *et al*., 1986)]. When the corresponding Mtap6 gene was cloned a decade later, it was realized that MAP6 proteins were sufficient to transfer cold resistance to microtubular networks in neuronal as well as non‐neuronal cells (Denarier *et al*., 1998b; Guillaud *et al*., 1998). From the multiple MAP6 isoforms produced by the Mtap6 gene (Denarier *et al*., 1998a), the highly expressed MAP6‐E (Embryonic) and MAP6‐N (Neuronal) are only found in the brain. In addition, it was shown that knocking down MAP6 protein expression in neuronal PC12 cells inhibited neurite formation upon differentiation (Guillaud *et al*., 1998). Hence, it was expected that MAP6 proteins possessed an obligatory function in microtubule stabilization in neuronal cells, possibly controlling axonal polarization and outgrowth. However, a MAP6 knockout (KO) mouse line was raised with success and hippocampal neurons cultured from these animals underwent every step of neuronal differentiation, including axonal polarization, full neuritic outgrowth and synaptogenesis (Andrieux *et al*., 2002). Gross brain organization also appeared to be normal although synaptic abnormalities were detected in MAP6 KO mice, such as depleted synaptic vesicle pools and defects in long‐term plasticity (Andrieux *et al*., 2002). These anomalies were associated with severe behavioural disorders including fragmentation of spontaneous activity, hyperlocomotion, anxiety‐related disorders, social withdrawal and deficient sensory gating mechanisms (Andrieux *et al*., 2002). Indications that MAP6 proteins may have biological roles unrelated to microtubules arose from observing their subcellular localization in the Golgi apparatus or in the mitochondria of non‐neuronal cells (Gory‐Faure *et al*., 2006, 2014). Also, phosphorylated MAP6‐N could be detected in the synaptic compartment of mature neuronal cultures (Baratier *et al*., 2006). More recently, it was shown that MAP6 proteins play a pivotal role in promoting axonal growth downstream of semaphorin3E signalling (Deloulme *et al*., 2015). Interestingly, a MAP6 mutant, unable to bind microtubules, was used to successfully restore semaphorin3E signalling in neuronal cultures, thus attributing to MAP6 proteins definitive non‐microtubular roles in neuronal development (Deloulme *et al*., 2015).

MAP6 KO animals displayed alterations in glutamate (Andrieux *et al*., 2002), dopamine (Brun *et al*., 2005) and serotonin (Fournet *et al*., 2010) neurotransmissions. Neurotransmission is a multistep procedure that follows action potential propagation to the presynaptic zone and results in releasing a specific neurotransmitter in the synaptic cleft. Voltage‐gated calcium channels (Ca_v_ according to their pore‐forming subunits) are critically involved in this process as they ensure calcium influx to the presynaptic axonal bouton, thereby triggering vesicular fusion with the plasma membrane, in a voltage‐ and calcium‐sensitive manner (Miller, 1987). The molecular family of Ca_v_ is composed of three main types – Ca_v_1, Ca_v_2 and Ca_v_3 – depending on their electrical properties and subcellular localization (Catterall *et al*., 2005). With the exception of Ca_v_3, all Ca_v_s are actually associated with ancillary subunits that contribute to their expression levels and modulate their activities. Modulation can be achieved by directly modifying the electrical properties of the functional subunits or by controlling various steps of their trafficking [see (Dolphin, 2016) for a recent review]. Other calcium channel effectors interact with the cytoplasmic C‐terminal tail of the pore‐forming subunit to modify its residence time in the plasma membrane, for example Mint/CASK (Maximov *et al*., 1999), calmodulin (Lee *et al*., 2000) or Tctex1 (Lai *et al*., 2005). Tctex1 had first been identified as a facultative light chain of brain cytoplasmic dynein (King *et al*., 1996), used for the apical transport of specific cargoes (Tai *et al*., 2001). Since, other biological roles in neurons have been attributed to Tctex1, independently from its interactions with dynein, for example in neuritic outgrowth or axon formation (Chuang *et al*., 2005), transport and egress of herpes simplex virus (Douglas *et al*., 2004), genesis of neurons from cortical precursor cells (Gauthier‐Fisher *et al*., 2009). In the present study, we demonstrate that MAP6 proteins interact with Tctex1 *in vitro* and are required for proper calcium signalling in hippocampal neurons.

## Materials and methods

### Yeast two‐hybrid

Baits were fusions of LexA‐BD with rat MAP6‐N fragments LNt, 5R and LCt (cDNAs cloned in pLexA, Addgene), and targets were fusions of Gal4‐AD fused to the polypeptides encoded by the cDNAs from the library, cloned in pAct2 (Clontech). First, L40 yeasts were separately transformed with the 3 pLexA plasmids encoding the baits, using Yeast Alkali‐cation kit (Bio 101) and selected on a medium without tryptophan. For the two‐hybrid screening, each of the three L40 yeast producing a bait was transformed with an amplified mouse brain cDNA library (Matchmaker CDNA library, Clontech), into which cDNAs are cloned in plasmid pACT2, using Yeast Alkali‐cation kit. Transfected yeasts were grown on a medium lacking tryptophan and leucine, and additionally selected by the lack of histidine in the medium, and by a colorimetric assay, involving the *HIS3* and *lacZ* reporter genes, respectively. For the colorimetric assay, colony‐lift filter assay was performed with nitrocellulose filters, liquid nitrogen freezing and a X‐gal solution, according to the manufacturer's protocol. Plasmids from the selected yeast clones were purified and sequenced to identify the target protein. For directed interactions, haploid yeast L40 (Mat a) or AMR70 (Mat α) were, respectively, transformed with pLexA plasmids encoding LexA‐BD fused to a MAP6‐N fragment, and with pAct2 plasmid encoding Gal4‐AD fused to mouse Tctex1. After mating of the haploid yeasts, selection was doubly performed as above.

### Animal research and breeding

The research involving animals was authorized by the ‘Direction Départementale de la protection des populations / Préfecture de l'Isère’ (Brocard, J., PhD, permit # 38 10 06) and by the ethics committee of the Grenoble Institute for Neuroscience, accredited by the French Ministry of Research. Homogeneous inbred C57BL6/129SvPas F1 mice were obtained by crossing MAP6 heterozygote 129SvPas male or female mice with MAP6 heterozygote C57BL6 female or male mice, respectively. Seventy‐five pregnant MAP6 heterozygote 129SvPas or C57BL6 females have been used for this study. Mice breeding was performed in compliance with French legislation and European Union Directive of 22 September 2010 (2010/63/UE).

### Neuronal cultures

Pregnant MAP6 heterozygote 129SvPas or C57BL6 females were euthanized by cervical dislocation and embryos swiftly shifted to phosphate‐buffered saline at room temperature. Then, hippocampi from E17.5 mice embryos were removed and digested with trypsin 2.5% (Invitrogen, France) diluted 1/10, at 37 °C for 15 min. After manual dissociation, hippocampal neurons from individual (Fig. 4B) or pooled embryos of the same genotype (Figs 2, 3, 4, 5) were plated onto one 60‐mm Petri dishes (BD Falcon, France) per hippocampus, containing 12 poly‐L‐lysine‐coated coverslips, in 5 mL DMEM 4.5 g/L + 10% horse serum (Invitrogen, France). Using cortices from the same embryos, it was also possible to plate 12 wells of a 24‐well Petri dish (BD Falcon, France) per embryo (Fig. 4D–E), in 0.5 mL D MEM 4.5 g/L + 10% horse serum. In both cases, the media were replaced by identical volumes of Neurobasal containing B‐27 supplement and glutamax (Invitrogen, France), about one hour and a half after plating [see (Andrieux *et al*., 2002) for more details].

One millilitre or 0.1 mL of fresh MACS medium (Miltenyi Biotec, France) containing B‐27 supplement, glutamax (Invitrogen, France) and 15 μm cytosine‐D‐arabinofuranoside (Sigma‐Aldrich, AraC; final concentration, 3 μm) was added every week to each 60‐mm Petri dish and each well of 24‐well Petri dish, respectively. In 60‐mm Petri dishes, an addition of 0.5 mL fresh MACS medium containing B‐27 supplement, glutamax but no AraC, was offset every week as well, for long‐term (> 10 days) cultures.

### Electrophysiology

Hippocampal neurons in culture after 6–7 days *in vitro* (DIV) were patch‐clamped and Ca^2+^ current was recorded at room temperature (22–24 °C) in whole‐cell configurations, in a bath medium containing: 5 mm BaCl_2_, 128 mm NaCl, 5 mm KCl, 1 mm MgCl_2_, 10 mm TEACl, 10 mm D‐glucose, 10 mm HEPES (pH 7.4 with NaOH), supplemented with 500 mm tetrodotoxin (Latoxan, France) and 10 μm nifedipine (Sigma‐Aldrich, France) to block Nav sodium channels and dihydropyridine‐sensitive Ca_v_1/L‐type calcium channels, respectively. Patch pipettes were filled with a solution containing: 110 mm CsCl, 3 mm Mg‐ATP, 0.5 mm Na‐GTP, 2.5 mm MgCl_2_, 10 mm EGTA‐Cs, 10 mm HEPES (pH 7.4 with CsOH) and had a resistance of 2–4 MΩ. All traces were corrected online for leak and capacitance currents, digitized at 10 kHz and filtered at 2 kHz, with an axopatch 200B (Molecular Devices, Sunnyvale CA, USA).

### Immunolabelling

Coverslips of hippocampal cultures after 3 DIV (Fig. 3B), 4 DIV (Figs 4C and 5C) or 8–9 DIV (Fig. 4B and D) were fixed for 25 min at 37 °C in PFS (4% paraformaldehyde + 4% sucrose) and permeabilized for 1 min in PBS + Triton X‐100 0.1%. When mentioned, fixation was preceded by a 15‐min incubation period with 5 μm Alexa‐546‐conjugated transferrin from human serum (T23364, Thermo Fisher Scientific, France, diluted to 10 mm in DMSO). Antibodies raised against microtubules [α3A1 homemade, mouse, 1/5000 (Paturle‐Lafanechere *et al*., 1994)], Ca_v_2.2/N‐type calcium channels (ACC‐002, rabbit, Alomone Labs, Israel, 1/300), Lamp1 (L1418, rabbit, Sigma‐Aldrich, France, 1/500), tyrosinated microtubules [YL1/2 homemade, rat, 1/5000 (Wehland & Willingham, 1983)] and/or tau (MAB3420, mouse, Millipore, 1/200), diluted in PBS + Tween‐20 0.1%, were used for a 1‐h incubation at room temperature. Coverslips were then rinsed in PBS + Tween‐20 0.1% and incubated for an additional 1 h with the appropriate species‐specific secondary antibody combined to Alexa‐488 (green, 1/500) (Thermo Fisher Scientific, France), Alexa‐546 (red, 1/1000) or Alexa‐647 (deep red, 1/500) (Jackson ImmunoResearch, Europe) at room temperature. Coverslips were then rinsed in PBS + Tween‐20 0.1%, in H_2_O and mounted in Dako Medium (Agilent Technologies, France) containing 1 μg/mL Hoechst 33258 (Sigma‐Aldrich, France). Images were taken with an inverted microscope Axioskop 50 (Carl Zeiss, France) controlled by metaview software (Universal Imaging, USA) using a 40 ×  oil immersion objective. Images were digitized using a Coolsnap ES camera (Roper Scientific, Trenton, NJ, USA). Image analysis was performed with the free imagej software (Schneider *et al*., 2012), as detailed in Supporting Information.

### Calcium imaging

Coverslips of hippocampal cultures after 8–9 DIV (Fig. 2) were incubated at 37 °C without CO_2_ in the presence of 1 μm Fluo‐4 (Thermo Fisher Scientific, France) in warm artificial cerebrospinal fluid [aCSF containing: 140 mm NaCl, 5 mm KCl, 1.5 mm CaCl_2_, 0.75 mm MgSO_4_, 1.25 mm NaH_2_PO_4_, 20 mm D‐glucose, 15 mm HEPES, pH 7.4 with NaOH] during 20–25 min. After 5–10 additional minutes in regular aCSF, each coverslip was mounted on a mini‐POC perfusion chamber (PeCon GmbH, Germany) and constantly perfused with 5 mL/min aCSF at room temperature. Imaging was performed as 1 image/6 s during 10–11 min, using an inverted microscope Axiovert 200M with a 20 ×  dry objective (Carl Zeiss, France) controlled by metaview software (Universal Imaging, USA). Metaview was set at multiple positions, enabling the simultaneous recording of one to four transfected neurons during the same experiment. When indicated, a KCl medium [containing: 100 mm NaCl, 45 mm KCl, 1.5 mm CaCl_2_, 0.75 mm MgSO_4_, 1.25 mm NaH_2_PO_4_, 20 mm D‐glucose, 15 mm HEPES, pH 7.4 with NaOH] replaced aCSF, in the absence or in the presence of 20 μm nimodipine (Sigma‐Aldrich, France, diluted to 20 mm in DMSO). Also, 1/3 NaCl was replaced with LiCl, both in the aCSF (93 mm NaCl + 47 mm LiCl) and KCl (67 mm NaCl + 33 mm LiCl) media when indicated. An additional 1 mm kynurenic acid (KA, Sigma‐Aldrich, France) was present when indicated as well.

For spontaneous activity recordings, coverslips of hippocampal cultures after 16–17 DIV (Fig. 3C) were incubated at 37 °C without CO_2_ in the presence of 2 μm Fluo‐4 (Thermo Fisher Scientific, France) in warm aCSF (see above) during 20–25 min. After five additional minutes in regular aCSF, each coverslip was mounted and constantly perfused with 2 mL/min of warm aCSF (> 30 °C). Imaging of a unique field was performed at 2 Hz during 8–10 min, using an inverted microscope Axiovert 200M with a 20 ×  dry objective (Carl Zeiss, France) controlled by metaview software (Universal Imaging, USA).

### Transfections

Coverslips of hippocampal cultures after 3 DIV (Figs 4C and 5C) or 7 DIV (Fig. 2E) were transferred to NMEM‐B27 medium (Minimal Essential Medium 1x, 1 mm sodium pyruvate, 15 mm HEPES, 2 mm L‐glutamine, B‐27 supplement 1x, 33 mm glucose, pH 7.4) just prior to adding 120 μL of calcium phosphate precipitate. Briefly, the precipitate was obtained by gently adding 2 μg of DNA as indicated below, in addition to 0.5 μg of a mCherry‐encoding plasmid (pmcherry‐C1, Clontech Laboratories, USA) or a GFP‐encoding plasmid (pEGFP‐N1, Clontech Laboratories, USA) and 250 mm CaCl_2_ in 60 μL of water, added dropwise to 60 μL of 2x BBS solution [50 mm BES, 280 mm NaCl, 1.5 mm Na_2_HPO_4_, pH 7.2 (Kingston *et al*., 2001)]. Neurons were then incubated at 37 °C with no CO_2_ for 90 min, washed three times with warm HEPES‐buffered saline solution [135 mm NaCl, 20 mm HEPES, 4 mm KCl, 1 mm Na_2_HPO_4_, 2 mm CaCl_2_, 1 mm MgCl_2_, 10 mm D‐Glucose, pH 7.3 (Kingston *et al*., 2001)] and their own initial medium after filtration (3 DIV) or fresh MACS medium (see above, 7 DIV) was added for a further 20‐ to 24‐h incubation at 37 °C with CO_2_.

### Plasmids

Numbering for rat MAP6‐N and MAP6‐E, mouse Tctex1 and human Ca_v_2.2/N‐type calcium channels is based on accession numbers NP_058900, CAA05555, NP_033368 and NP_000709, respectively. All constructs were sequence‐verified.

For two‐hybrid experiments, cDNAs encoding MAP6‐N baits LNt (aa 1–225), 5R (aa 221–455), LCt (aa 451–952), LCt_451‐572_ (aa 451–572), LCt_451‐572 AAAIA_ (aa 451–572, with _532_AAAIA_536_ mutation), LCtΔMn3 (aa 451–952, with aa 481–495 deletion), LCt_AAAIA_ (aa 451–952, with _532_AAAIA_536_ mutation) and LCt ΔMn3_AAAIA_ (aa 451–952, with aa 481–495 deletion and with _532_AAAIA_536_ mutation) were cloned in pLexA (Addgene) with a LexA‐BD N‐terminal fusion, whereas cDNA encoding target Tctex1 (all 113 aa) was cloned in pAct2 (Addgene) with a Gal4‐AD N‐terminal fusion.

For immunoprecipitation experiments or to transfect neurons *in vitro*, cDNAs encoding MAP6‐N (all 952 aa) and mutants LCt (aa 451–952), LCtΔMn3 (aa 451–952, with aa 481–495 deletion), LCt_AAAIA_ (aa 451–952, with _532_AAAIA_536_ mutation) and LCt ΔMn3_AAAIA_ (aa 451–952, with aa 481–495 deletion and with _532_AAAIA_536_ mutation) were cloned in pSG5 (Stratagene); cDNAs encoding MAP6‐E (all 660 aa) and mutants MAP6‐EΔ1 (aa 2–19 deletion), MAP6‐EΔMn3_AAAIA_ (aa 527–541 deletion and _578_AAAIA_582_ mutation) and MAP6‐EΔ1ΔMn3_AAAIA_ (aa 2–19 deletion, aa 527–541 deletion and _578_AAAIA_582_ mutation) were cloned in pcDNA3.1(+) (Invitrogen, France); cDNA encoding Tctex1 (all 113 aa) was cloned in pEGFP‐N1 (Clontech) to encode for Tctex1‐GFP; cDNA encoding Tctex1 (all 113 aa) and NCD4 [Ca_v_2.2/N‐type channel aa 2020–2164 (Lai *et al*., 2005)] was cloned in pcDNA3.1(‐)/myc‐His‐A (Invitrogen, France), with myc and 6 His C‐terminal tags.

### Automatic plate reading

Twenty‐four‐well plates of cortical cultures after 7–8 DIV were incubated at 37 °C without CO_2_, in the presence of 1 μm Fluo‐4 (Thermo Fisher Scientific, France) in warm aCSF containing LiCl + KA, in the absence [nimo] or presence [toxins] of 20 μm nimodipine (see above, Calcium imaging), during 20 min. Each well was rinsed with aCSF containing LiCl + KA, without [nimo] or with [toxins] 20 mm nimodipine and 450 μL of fresh medium containing also DMSO or 20 μm nimodipine [nimo] on the one hand or DMSO, 320 nm ω‐conotoxin GVIA (STC‐750, Alomone Labs, Israel, diluted to 160 μm in DMSO) or 180 nm ω‐agatoxin IVA (STC‐750, Alomone Labs, Israel, diluted to 90 μm in DMSO) [toxins] on the other hand, was added to the corresponding wells. After an additional 5‐min incubation at 37 °C without CO_2_, entire plates were transferred to a Pherastar automatic plate reader (BMG Labtech, Germany) set to 37 °C, to record fluorescence intensity at 2 Hz during 30 s, for each well, as described at http://www.bmglabtech.com/media/35216/1043854.pdf. After 10 s, an automatic injection of 50 μL KCl 500 mm (final concentration, 50 mm) stimulated calcium influx.

### Immunoprecipitation experiments

On the one hand, COS‐7 cells in P100 Petri dishes were transfected with plasmids encoding Tctex1‐GFP and MAP6‐N or Tctex1‐myc and LCt mutants using Lipofectamine reagent (Thermo Fisher Scientific, France). One day after transfection, cells were rinsed with PBS and lysed in 400 μL [Tris 50 mm, NaCl 150 mm, DOC 0.1%, Triton X‐100 1%, glycerol 10%, pH 8.0, in the presence of protease inhibitors (Complete Cocktail tablets, Roche)] to produce the cell lysate (CL) fraction, after performing an additional 2‐min centrifugation at 10 000 ***g*** to discard cellular debris. Cell lysates were then incubated for 2.5 h at 4 °C with Sepharose beads (GE Healthcare, France) coupled with either protein G + rabbit anti‐GFP (Life Technologies, Gaithersburg, USA) or anti‐MAP6 antibodies [23N + 23C (Guillaud *et al*., 1998)] or protein A+mouse anti‐myc antibody (sc‐40, Santa Cruz Biotechnology Inc., Germany), before performing a 3‐min centrifugation at 10 000 ***g*** to collect the beads. After ample washing, complexes were separated by electrophoresis, blotted and revealed using the following antibodies: rabbit anti‐GFP (1/1000, Life Technologies, Gaithersburg, USA), mouse anti‐MAP6‐N [175, homemade, 1/2000 (Pirollet *et al*., 1989)] or rabbit anti‐Tctex1 [2025 + 2026, homemade, 1/2000 each, see Supporting Information].

On the other hand, HEK‐293 T17 cells from ATCC were transfected with plasmids encoding Tctex1‐GFP and MAP6‐E mutants (see above) or NCD4‐myc and MAP6‐E mutants (see above) or Tctex1‐GFP, NCD4‐myc and MAP6‐E mutants using a calcium phosphate kit (Life Technologies, Gaithersburg, USA). One day after transfection, cells were rinsed with PBS and lysed in the presence of protease inhibitors (Complete Cocktail tablets, Roche). For Tctex1‐GFP and MAP6‐E mutant transfection, the lysis buffer was 50 mm Tris pH8, 15 mm NaCl, 1% NP40, 0.5% sodium deoxycholate, 0.1% SDS; for NCD4‐myc and MAP6‐E mutant transfection, the lysis buffer was 20 mm Tris, 1 mm EGTA, 1 mm EDTA, 5 mm NaF, 1 mm DTT, 2 mm Na_3_VO_4_, 0.27 m sucrose, 0.5 % Triton X‐100, pH 7.2; for Tctex1‐GFP, NCD4‐myc and MAP6‐E mutants transfected altogether, lysis buffer was 137 mm NaCl, 2.7 mm KCl, 4.3 mm Na2HPO4, 1.4 mm KH2PO4, 5 mm EDTA, 5 mm EGTA, 0.05 % Triton X‐100. After centrifugation of the cell lysate at 10 000 ***g*** for 20 min at 4 °C, the supernatant was incubated O/N at 4 °C in the presence of Sepharose beads (GE Healthcare, France) coupled with protein G + rabbit anti‐MAP6 antibody [23N (Guillaud *et al*., 1998)]. Complexes were precipitated by 40 μL Dynabeads Protein G (Invitrogen, France) for 40 min at RT. The immunoprecipitates were washed six times with lysis buffer. Complexes were separated by electrophoresis, blotted and revealed using the following antibodies: rabbit anti‐GFP (1/3000, Invitrogen, France), mouse anti‐myc (1/3000, AM1007a, Abgent, San Diego, CA, USA) and 23N rabbit anti‐MAP6 (1/3000).

### Quantitative Western Blots from brain extracts

Brains regions from 3‐week‐old WT and MAP6 KO mice littermates were dissected out, and whole extracts from cerebellum, hippocampus and cortex were prepared by mechanical trituration in RIPA buffer (25 mm Tris‐HCl, 150 mm NaCl, 1% NP‐40, 1% Na‐deoxycholate, 0.1% SDS, pH 7.6) followed by a 2‐min centrifugation at 10 000 ***g*** to discard large debris. Brain extracts migrated on SDS‐PAGE gels were transferred onto nitrocellulose membranes; membranes were then cut to enable immunodetection of Ca_v_2.2/N‐type calcium channels (ACC‐002, rabbit, Alomone Labs, Israel, 1/500) and neuron‐specific enolase (AB9698, chicken, Merck Millipore, France, 1/2500), a MAP6‐insensitive marker, from the same extract. Quantification of Ca_v_2.2/N‐type channels contents from each brain region of each animal was expressed as mean Sqr (Ca_v_2.2/NSE) ratios from one to three independent Western blottings.

### Statistical analysis

Statistics were performed using the prism 5.0 software (GraphPad Software, San Diego, CA, USA). Throughout the study, raw data were compared using nonparametric Mann–Whitney *t*‐tests (Figs 2C and D, and 3B) or Kruskal–Wallis anova comparisons (Fig. 2E) while normalized data were compared using parametric Student *t*‐tests (Figs 2A, 3C and 4) or one‐way anova comparisons (Fig. 5C). The results are presented as mean ± standard error.

## Results

### Tctex1 interacts with MAP6 proteins

**Figure 1:**
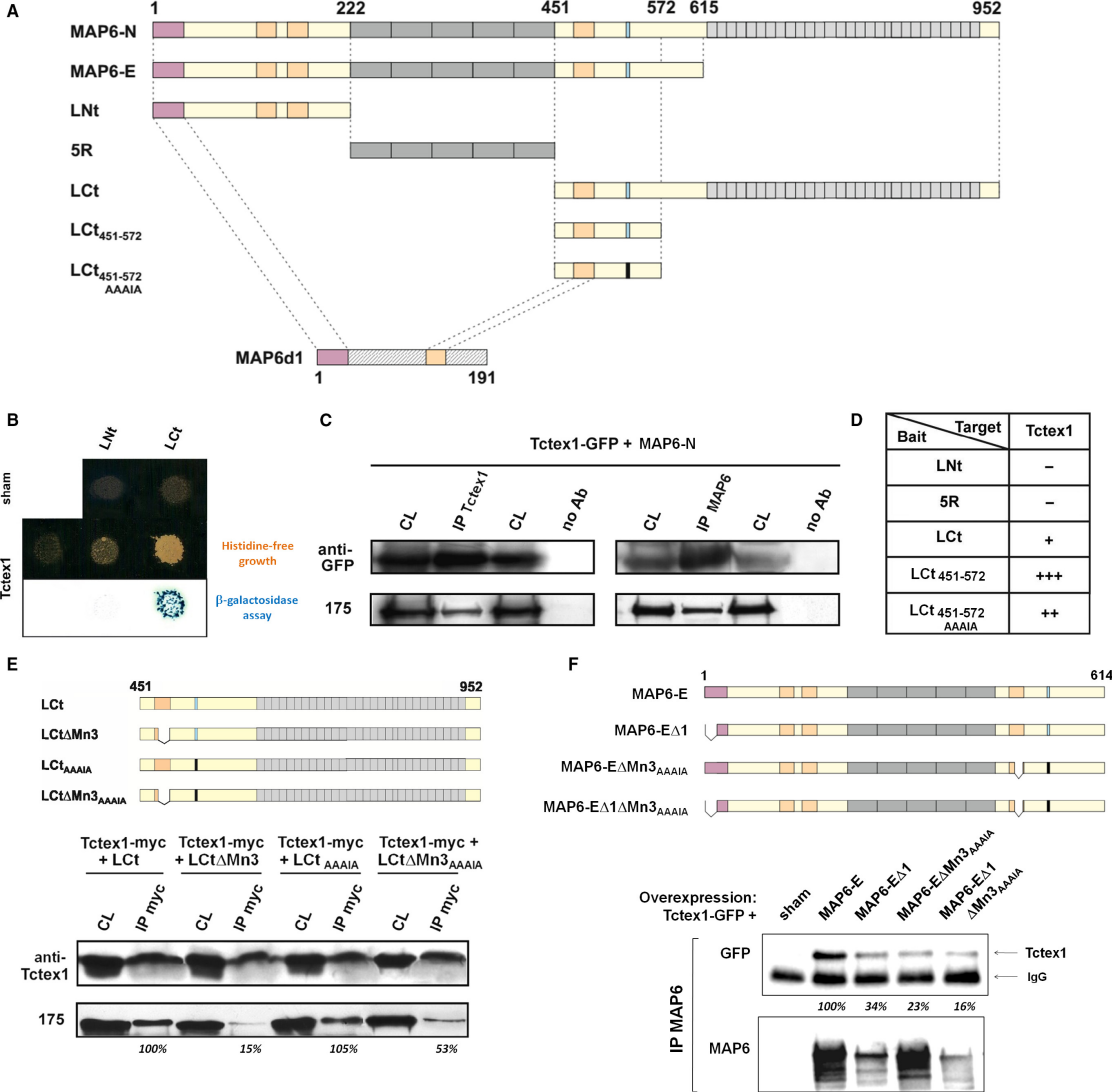
Tctex1 interacts with MAP6 proteins. (A) Schematic representation of rat neuronal MAP6 proteins (MAP6‐N, MAP6‐E and MAP6d1) and the MAP6 fragments used for two‐hybrid screenings. Note that the N‐terminal (LNt), central (5R) and C‐terminal (LCt) fragments are complementary. Coloured modules: mauve, palmitoylable N‐terminal domain (Gory‐Faure *et al*., 2006); light orange: MT‐stabilizing domains (Bosc *et al*., 2001); dark grey: thermo‐sensitive MT‐binding domains (Delphin *et al*., 2012); light grey: 28 C‐terminal repeats (Bosc *et al*., 2003); light blue: consensus Tctex1‐binding sequence RRRIR (Mok *et al*., 2001); black: AAAIA‐mutated corresponding sequence; hatched: MAP6d1‐specific aa with no homology to MAP6 proteins. The aa numbers are noted above. (B) Growth selection of yeasts containing the only baits LNt or LCt (top row) or a combination of the Tctex1 prey and either bait, either on histidine‐free medium (middle row) or visualization of protein interaction by staining of β‐galactosidase activity (bottom row). (C) Immunoprecipitation of protein complexes obtained from COS‐7 cells transfected with plasmids encoding Tctex1‐GFP and MAP6‐N, using sepharose beads coupled to either an anti‐GFP antibody or a mix of anti‐MAP6 antibodies (23N and 23C). Cell lysates (CL) and immunoprecipitated protein complexes (IP) were then analysed by SDS‐PAGE and Western blotting, using an anti‐GFP antibody (to evidence Tctex1) or an anti‐MAP6 antibody (175, to evidence MAP6). Control experiments were performed with no antibody coupled to the beads (no Ab). (D) Summary table of the two‐hybrid interactions assessed from growth on histidine‐free medium and staining of β‐galactosidase activity as described in (B). +++: very fast growth and strong staining; ++: medium growth and staining; +: slow growth and weak staining; ‐: no growth nor staining. (E) Schematic representation of MAP6‐N C‐termini (LCt, upper panel) used for immunoprecipitation with Tctex1‐myc (lower panel). Colour code is presented as in (A). Each immunoprecipitation was performed with an anti‐myc antibody and revealed with an immunoblot antibody against Tctex1 (Antibodies 2025 and 2026, see Supporting Information) or an anti‐MAP6 (175) to evidence MAP6‐N C‐termini. MAP6‐related bands were quantified, background subtracted and normalized by that obtained with non‐mutated LCt (italic percentages). (F) Schematic representation of full‐length MAP6‐E or MAP6‐E mutants Δ1, ΔMn3_AAAIA_ or Δ1ΔMn3_AAAIA_ (lower panel) used for immunoprecipitation with Tctex1‐GFP (upper panel). Colour code is presented as in (A). Each immunoprecipitation was performed with the anti‐MAP6 23N antibody and immunorevealed by Western blotting against GFP or MAP6 (23N). Note that the many MAP6 bands detected after immunoprecipitation correspond to specific degradation products as there is no signal in the sham‐transfected condition. The contaminating IgG band, close to that of Tctex1‐GFP, is indicated. Tctex1‐related bands were quantified, background subtracted and normalized by that obtained with non‐mutated MAP6‐E (italic percentages). [Colour figure can be viewed at wileyonlinelibrary.com].

To identify MAP6 partner proteins, we performed a yeast two‐hybrid screening of a mouse brain cDNA library (Matchmaker CDNA library, Clontech) using several fragments of MAP6‐N as bait, divided into N‐terminal (LNt), central (5R) and C‐terminal (LCt) fragments as shown Fig. 1A. Amongst the clones pulled out by the LCt fragment, three were identical to Tctex1, a light chain subunit of the dynein motor protein complex (King *et al*., 1996). The interaction between Tctex1 and LCt was assessed by growth on histidine‐deficient medium and by a colorimetric assay, involving the *HIS3* and *lacZ* reporter genes, respectively (Fig. 1B). In contrast to the LCt fragment, the LNt fragment did not bind Tctex1 (Fig. 1B). We further tested the selective interaction between Tctex1 and MAP6‐N by performing co‐immunoprecipitations experiments with lysates from transfected COS7 cells expressing Tctex1‐GFP and MAP6‐N. When the immunoprecipitation was performed with an anti‐GFP antibody, MAP6‐N was found associated with Tctex1‐GFP. When the immunoprecipitation was performed using anti‐MAP6 antibodies (23N/23C, see Material & Methods), Tctex1‐GFP was detected together with MAP6‐N (Fig.1C). To localize more precisely the domains of MAP6 involved in the interaction with Tctex1, we engineered several truncations of the LCt fragment fused to the LexA domain (Fig. 1A). The fragment corresponding to aa 451–572 displayed the strongest interaction with Tctex1 (Fig. 1D). Within this fragment, a RRRIR stretch of aa at positions 532–536 was matching the R/K‐R/K‐X‐X‐R/K consensus sequence for Tctex1 binding (Mok *et al*., 2001). Using peptide spots technology, we determined that the RRRIR sequence of MAP6 was indeed able to bind to recombinant GST‐Tctex1 (Fig. S1). However, when the RRRIR sequence was mutated into AAAIA, the interaction with Tctex1 was still strong in the yeast (Fig. 1D). Performing immunoprecipitation experiments again, we confirmed that only mutating the consensus sequence was not enough to abolish the interaction between the LCt fragment of MAP6‐N and Tctex1 (Fig. 1E). In parallel experiments, we have shown that the distantly related MAP6d1 protein (Gory‐Faure *et al*., 2006) was also able to immunoprecipitate Tctex1 (Fig. S2). As MAP6 proteins display a modular organization, we hypothesized that the sequences highly homologous between MAP6 proteins and MAP6d1 – namely the 83% homologous N‐terminal stretch and a 71% homologous microtubule‐stabilizing domain (Fig. 1A) – were likely to be involved in the interaction with Tctex1. Indeed, further deleting the microtubule‐binding domain Mn3 [Δaa481–495 (Bosc *et al*., 2001)] of the LCt fragment had a significant effect by bringing the interaction close to zero (Fig. 1E). For further studies, we used the full‐length isoform MAP6‐E instead of MAP6‐N as the C‐terminal repeats did not seem to have an impact on the interaction with Tctex1 (Fig. 1F). Mutating the only N‐terminus of MAP6‐E (MAP6‐EΔ1, Fig. 1F) displayed about the same decrease in Tctex1 co‐immunoprecipitation as the combination of Mn3 deletion and AAAIA mutation (MAP6‐EΔMn3_AAAIA_, Fig. 1F). Combining these two modifications (MAP6‐EΔ1ΔMn3_AAAIA_, Fig. 1F) decreased the interaction even further. Hence, the interaction surface of MAP6 with Tctex1 seems to be complex, with aa from the N‐terminus, the Mn3 domain and the R/K‐R/K‐X‐X‐R/K consensus sequence likely to participate.

### MAP6 is required for proper calcium signalling

**Figure 2:**
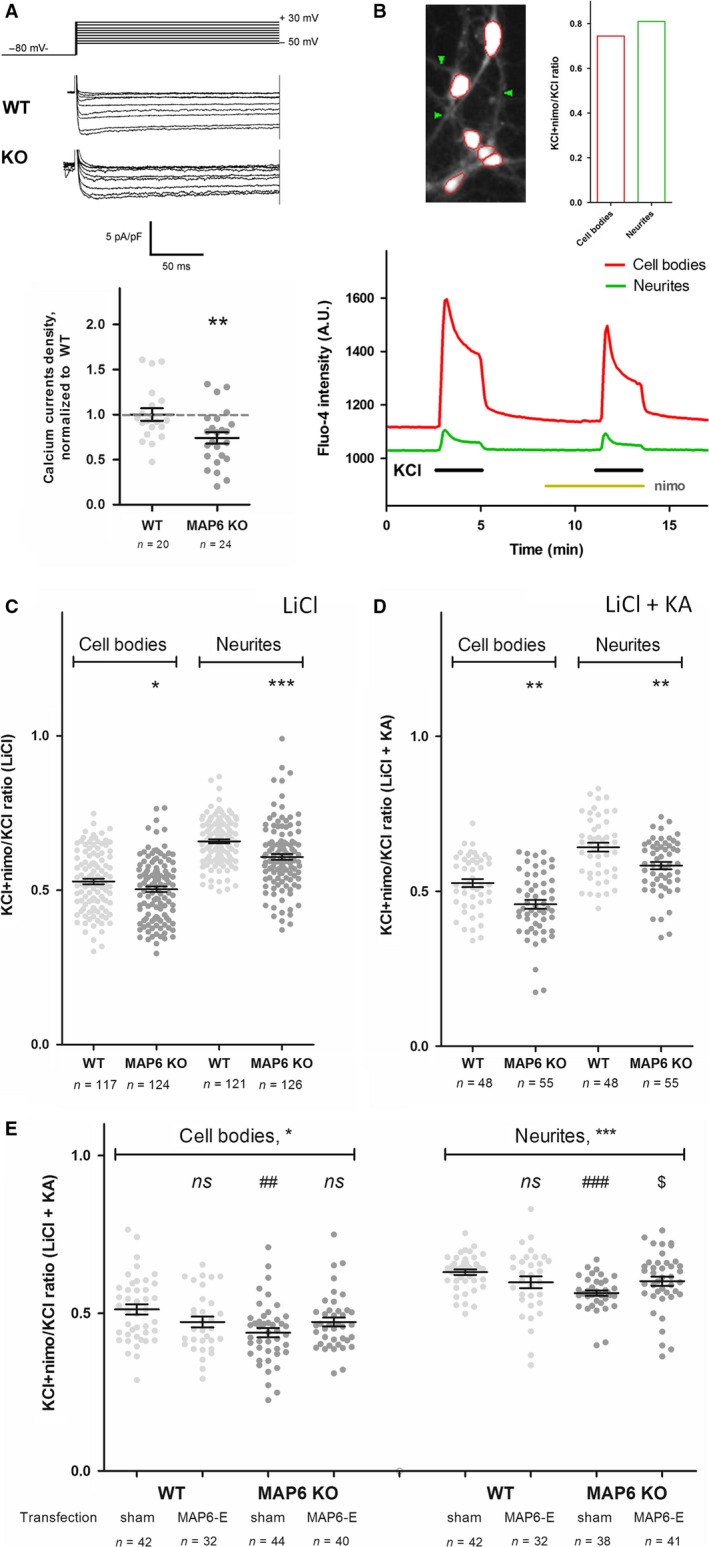
MAP6 KO neurons display deficient calcium signalling, rescued by MAP6‐E expression. (A) Superimposed current density traces obtained in whole‐cell configuration: the holding potential was −80 mV and cells were depolarized from −50 mV to +30 mV, by steps of 10 mV (upper panel). Quantification of whole‐cell patch‐clamp recordings of WT (white discs) and MAP6 KO (grey discs) hippocampal neurons, normalized by that of WT neurons for each neuronal culture (lower panel, dashed grey line = 100%). *n* represents the total number of neurons measured from four independent neuronal cultures. **, *P *<* *0.01 as compared to WT, using an unpaired parametric *t*‐test. (B) Example of a recorded field of WT hippocampal neurons from which cell bodies (circled in red) and neurites (green arrowheads) were detected and used for fluorescence intensity measurements, as indicated on the bottom panel (red line, cell bodies; green line, neurites). The histogram on the right panel corresponds to the quantification of the peak ratios obtained in the presence of KCl + nimodipine, divided by that obtained in the presence of KCl alone, for both regions (red column, cell bodies; green column, neurites). The ratios represent the fraction of the non‐Ca_v_1/L‐type calcium channels in the initial calcium peak. (C) Quantification of the peak ratios obtained in cell bodies and neurites of WT (white discs) and MAP6 KO (grey discs) hippocampal neurons using aCSF medium where 1/3 NaCl was replaced with LiCl. *n* represents the total number of fields recorded from five independent neuronal cultures. *, *P *<* *0.05 and ***, *P *<* *0.001, as compared to the corresponding WT, using unpaired nonparametric *t*‐tests. (D) Quantification of the peak ratios obtained in cell bodies and neurites of WT (white discs) and MAP6 KO (grey discs) hippocampal neurons using aCSF medium where 1/3 NaCl was replaced with LiCl and in the presence of 1 mm Kynurenic Acid. *n* represents the total number of fields recorded from three independent neuronal cultures. **, *P *<* *0.01, as compared to the corresponding WT, using unpaired nonparametric *t*‐tests. (E) Quantification of the peak ratios obtained in cell bodies and neurites of transfected WT (white discs) and MAP6 KO (grey discs) hippocampal neurons, using aCSF medium where 1/3 NaCl was replaced with LiCl and in the presence of 1 mm kynurenic acid as in (D). Each transfected cell was detected via mCherry expression while the accompanying plasmid was unlabelled (sham = vector alone or MAP6‐E). *n* represents the total number of transfected cells recorded from three independent neuronal cultures. *, *P *<* *0.05 and ***, *P* < 0.001 using a nonparametric one‐way anova, followed by Dunn's multiple comparison tests : ns, not significantly different from the corresponding sham‐transfected cells, ##, *P *<* *0.01 and ###, *P *<* *0.001 as compared to sham‐transfected WT, $, *P *<* *0.05 as compared to sham‐transfected KO. [Colour figure can be viewed at wileyonlinelibrary.com].

In hippocampal neurons, Tctex1 participates in the surface expression of calcium channels (Lai *et al*., 2005). More specifically, preventing the direct interactions of Tctex1 with calcium channels was reported to decrease Ca_v_2‐type calcium currents (Lai *et al*., 2005). As MAP6 also interacts with Tctex1 *in vitro*, we intended to address MAP6 contribution to Ca_v_2‐mediated calcium signalling in neuronal cultures (Fig. 2). Electrophysiological recordings of hippocampal neurons revealed a global ~25% decrease in calcium current densities in MAP6 KO vs. WT neurons (Fig. 2A, 0.740 ± 0.063 vs. 1.000 ± 0.068, *P *=* *0.008). To access spatial resolution, neuronal cultures were also loaded with the calcium‐sensitive fluorescent dye fluo‐4 and stimulated with KCl, in the absence or in the presence of nimodipine, a Ca_v_1/L‐type calcium channel inhibitor. Using this paradigm, we hypothesized that the (KCl+nimo/KCl) calcium peak ratio would represent the net contribution of Ca_v_2‐type calcium channels to increases of cytoplasmic calcium during KCl stimulations (Fig. 2B). However, much larger ratios than expected were measured [> 0.75 vs. 0.5 as described before at this stage (Pravettoni *et al*., 2000)], thus indicating that other major actors of calcium signalling were stimulated in the presence of KCl. Therefore, we replaced a fraction of NaCl with LiCl to inhibit the Na^+^/Ca^2+^ exchanger (Khodorov *et al*., 1999), thus leading to measure significantly smaller peak ratios in MAP6 KO vs. WT neurons, both in the cell bodies and neurites (Fig. 2C, 0.503 ± 0.009 vs. 0.529 ± 0.009 in cell bodies, *P *=* *0.043 and 0.608 ± 0.009 vs. 0.659 ± 0.007 in neurites, *P *<* *0.001). Note that the larger ratios recorded in neurites are consistent with a higher concentration of Ca_v_2‐type channels there than in cell bodies (Pravettoni *et al*., 2000). Adding kynurenic acid (KA), an inhibitor of NMDA receptors, to the recording medium further unmasked the apparent deficit of Ca_v_2‐type channels in the cell bodies of MAP6 KO neuronal cultures (Fig. 2D, 0.458 ± 0.014 vs. 0.527 ± 0.013 in cell bodies, *P *=* *0.001 and 0.583 ± 0.012 vs. 0.642 ± 0.012 in neurites, *P *=* *0.005). In order to establish that MAP6 was sufficient to restore calcium channel activity in MAP6 KO neurons, MAP6‐E expression was rescued before measuring calcium imaging. For each experiment, neurons of both genotypes – MAP6 KO and WT – were transfected with a mCherry‐expressing plasmid to detect the transfected cells, as well as a MAP6‐E‐ or sham‐encoding plasmid. MAP6‐E rescue had no significant effect on calcium signalling of WT or MAP6 KO cell bodies, but it did significantly increase the (KCl+nimo/KCl) calcium peak ratio in neurites of MAP6 KO neurons specifically (Fig. 2E, 0.602 ± 0.015 vs. 0.564 ± 0.009, *P *<* *0.05 as compared to neurites from sham‐transfected MAP6 KO neurons). Hence, calcium signalling mediated by Ca_v_2‐type calcium channels is deficient in MAP6 KO neurons and may be rescued by exogenous MAP6‐E expression.

### Functional Ca_v_2.2/N‐type calcium channels are deficient in MAP6 KO neurons

**Figure 3:**
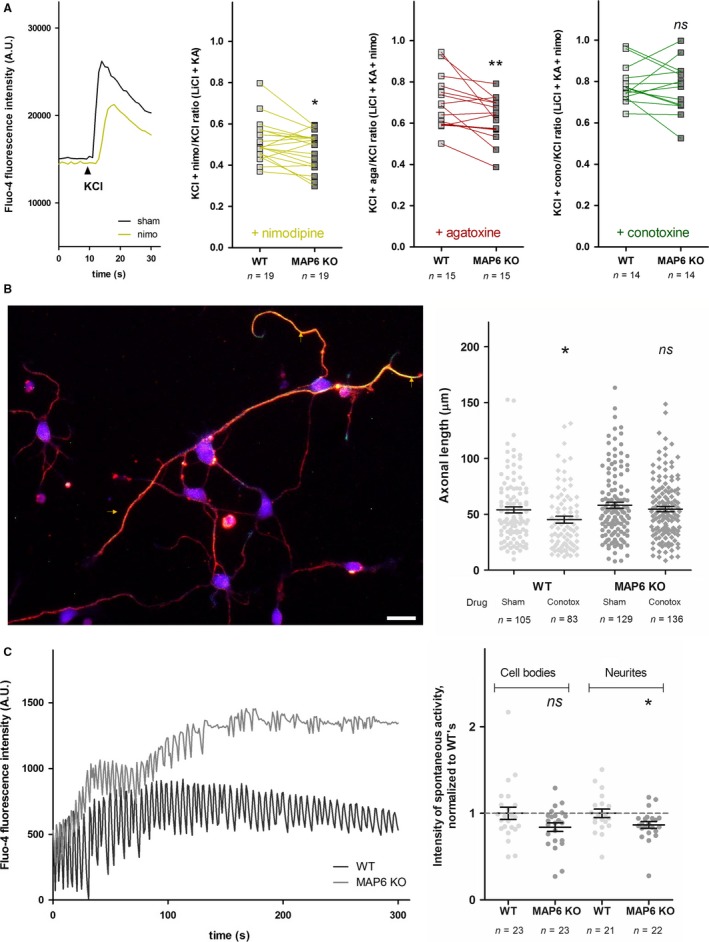
Specificity of Ca_v_2‐type calcium channels deficit in MAP6 KO neurons. (A) Left panel, examples of KCl‐stimulated fluo‐4‐loaded WT cortical neurons in the absence (black line) or presence (yellow line) of 20 μm nimodipine. Right panels, ratios of KCl‐elicited fluorescence intensity peaks, recorded from WT (white squares) and MAP6 KO (grey squares) cortical neurons in the presence or absence of 20 μm nimodipine (yellow curves), 180 nm ω‐agatoxin IVA (red curves) or 320 nm ω‐conotoxin GVIA (green curves). *n* represents the total number of wells recorded from eight independent neuronal cultures. ns, *P* > 0.05, *, *P *<* *0.05 and **, *P *<* *0.01 against the corresponding WT, using paired nonparametric *t*‐tests. Note that Wilcoxon *t*‐tests have been performed to account for technical data pairing between neighbouring MAP6 KO and WT culture wells and this pairing was deemed as highly significant (*P *<* *0.01) for each panel. (B) Left panel, example of identified axons (yellow arrowheads) from a neuronal culture with immunolabelled microtubules (red), tau (green) and nuclei (blue). Right panel, measurements of axonal length of WT (white symbols) and MAP6 KO (grey symbols) hippocampal neurons after 48 h in the absence (sham, discs) or presence (conotox, diamonds) of 320 nm ω‐conotoxin GVIA. *n* represents the total number of neurons measured from three independent neuronal cultures. ns, *P *>* *0.05 and *, *P *<* *0.05 as compared to corresponding sham, using unpaired nonparametric *t*‐tests. Scale bar = 10 μm. (C) Left panel, examples of spontaneous calcium activity recorded from fluo‐4‐loaded WT (black line) and MAP6 KO (grey line) hippocampal neurons. Right panel, quantification of mean peak intensity during spontaneous calcium activity of fluo‐4‐loaded WT (white discs) and MAP6 KO (grey discs) hippocampal neurons, recorded in cell bodies and neurites, independently, normalized by that of WT (dashed grey line = 100%). *n* represents the total number of fields recorded from six independent neuronal cultures. ns, *P *>* *0.05 and *, *P *<* *0.05 as compared to corresponding WT, using unpaired parametric *t*‐tests.[Colour figure can be viewed at wileyonlinelibrary.com].

Members of the Ca_v_2‐type calcium channel family may be inhibited by very specific natural toxins purified from cone snails' or spiders' venom: ω‐agatoxin IVA against Ca_v_2.1/PQ‐type, ω‐conotoxin GVIA against Ca_v_2.2/N‐type and SNX‐482 against Ca_v_2.3/R‐type (Catterall *et al*., 2005). In cortical cultures after 6–8 days *in vitro*, Ca_v_‐mediated calcium signalling is expected to be mostly supported by Ca_v_2.1 and Ca_v_2.2 (Schlick *et al*., 2010). Hence, we used cortical cultures grown on 24‐well plates to study the contribution of Ca_v_2‐type calcium channels to KCl‐elicited calcium signalling in MAP6 KO neurons. Each well was loaded with the calcium‐sensitive fluorescent dye fluo‐4, and a KCl stimulation was performed in the presence or absence of specific toxins (Fig. 3A). As a control, adjacent wells were stimulated in the absence or presence of nimodipine and the corresponding ratios, obtained from WT and MAP6 KO cultures, were paired and compared (yellow curves, 0.515 ± 0.023 vs. 0.473 ± 0.021, *P *=* *0.031). In the presence of ω‐agatoxin IVA, a significant difference of ratios was observed (red curves, 0.698 ± 0.033 vs. 0.622 ± 0.027, *P *=* *0.007 for WT and MAP6 KO, respectively) whereas none was detected in the presence of ω‐conotoxin GVIA (green curves, 0.784 ± 0.025 vs. 0.769 ± 0.033, *P *=* *0.583 for WT and MAP6 KO, respectively). This suggested that diminished Ca_v_2.2/N‐type calcium channels were likely to underlie the decreased calcium signalling in MAP6 KO neurons. We performed complementary measures from functional assays to assess biological features strongly related to either Ca_v_2.1/PQ‐ or Ca_v_2.2/N‐type calcium channels in neuronal cultures.

In developing neurons, Ca_v_2.2/N‐type channels have been associated with axonal specification and growth (Pravettoni *et al*., 2000). Hence, we measured axonal growth from MAP6 KO and WT neurons in the presence of the ω‐conotoxin (Fig. 3B). After a 48‐h treatment, axonal length was significantly diminished in WT neurons, in the presence of ω‐conotoxin, as compared to sham‐treated cultures (Fig. 3B, 45.2 ± 3.1 μm vs. 54.0 ± 2.8 μm, *P *=* *0.013). In contrast, axonal growth of MAP6 KO neurons displayed no sensitivity to the toxin (Fig. 3B, 54.7 ± 2.3 μm vs. 58.1 ± 2.8 μm, *P *=* *0.577). This demonstrated that functional Ca_v_2.2/N‐type calcium channels in MAP6 KO neurons must be missing in the plasma membrane, thereby not supporting axonal growth as pre‐eminently as they do in WT neurons.

**Figure 4:**
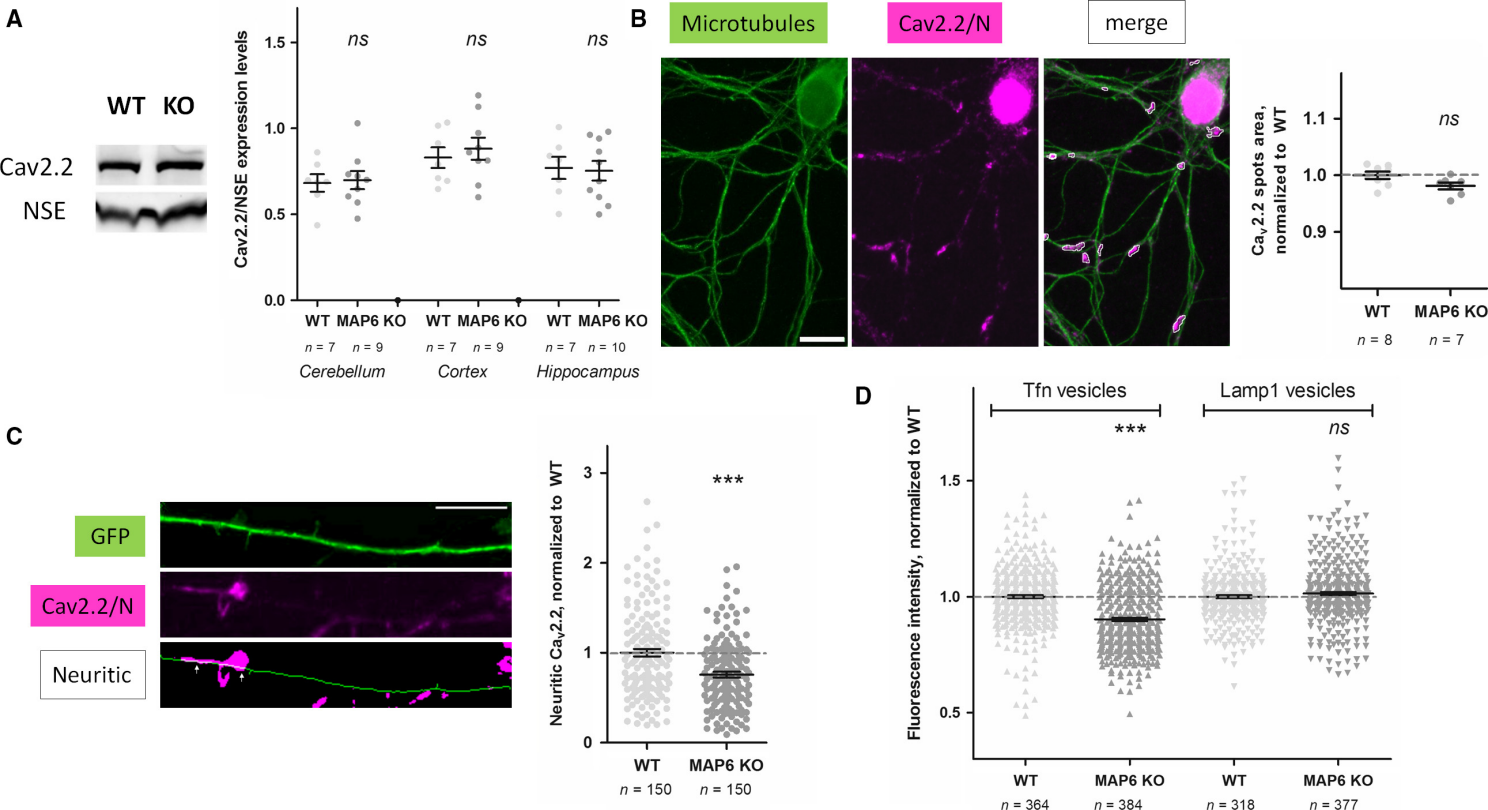
Calcium channel expression and traffic in MAP6 KO neurons. (A) Left panels, example of Western blotting of Ca_v_2.2/N‐type calcium channels and Neuron‐specific enolase (NSE) obtained from WT and MAP6 KO hippocampal extracts. Right panel, quantification of Ca_v_2.2/N‐type channels normalized to NSE ratios obtained from cerebella, cortices and hippocampi of WT (white discs) and MAP6 KO (grey discs) animals. ns, *P *>* *0.05 against the corresponding WT, using unpaired parametric *t*‐tests. (B) Left panels, immunolabelling of microtubules (green) and Ca_v_2.2/N‐type calcium channels (magenta) and detection of spots on a merged image using ImageJ (see Supporting Information for details). Scale bar = 10 μm. Right panel, quantification of Ca_v_2.2/N‐type calcium channels from WT (white discs) and MAP6 KO (grey discs) hippocampal neurons, normalized by that of WT neurons (dashed grey line = 100%) for each neuronal culture. *n* represents the total number of individual embryos used during four independent preparations of neuronal cultures. ns, *P *>* *0.05 as compared to WT, using an unpaired parametric *t*‐test. (C) Left panel, example of a neurite from a GFP‐transfected WT neuron (green) with Ca_v_2.2/N‐type calcium channels immunodetection (magenta) and measurement of neuritic content on a merged image using ImageJ (white, see Supporting Information for details). Scale bar = 10 μm. Right panel, quantification of neuritic Ca_v_2.2/N‐type calcium channels content from GFP‐transfected WT (white discs) and MAP6 KO (grey discs) hippocampal neurons, normalized by that of WT neurons (dashed grey line = 100%). *n* represents the total number of transfected neurons measured from three independent neuronal cultures. ***, *P *<* *0.001 as compared to WT, using an unpaired parametric *t*‐test. (D) Quantification of Tfn vesicles (triangles up) and Lamp1 vesicles (triangles down) from WT (white triangles) or MAP6 KO (grey triangles) hippocampal neurons, normalized by that of WT neurons (dashed grey line = 100%). *n* represents the total number of neurons measured from four independent neuronal cultures. ns, *P *>* *0.05 and ***, *P *<* *0.001 as compared to the corresponding WT, using unpaired parametric *t*‐tests. [Colour figure can be viewed at wileyonlinelibrary.com].

In mature neuronal networks, Ca_v_2.1/PQ‐type calcium channels were shown to be essential for spontaneous neurotransmitter release (Qian & Noebels, 2001). We loaded mature MAP6 KO and WT hippocampal cultures after 16–17 days *in vitro* with the calcium‐sensitive fluorescent dye fluo‐4 to measure spontaneous calcium activity (Fig. 3C). The number of observed calcium peaks showed great variability, and no significant difference of frequency was observed between MAP6 KO and WT neurons (not shown). In contrast, peak intensity was significantly smaller in MAP6 KO neurons as compared to WT (Fig. 4C, 0.840 ± 0.050 vs. 1.000 ± 0.072, *P *=* *0.074 in cell bodies and 0.867 ± 0.039 vs. 1.000 ± 0.049, *P *=* *0.038 in neurites, respectively). These results indicated that Ca_v_2.1/PQ‐type calcium channels may be diminished in mature MAP6 KO neuronal networks, thus mediating less intense spontaneous activity than in WT neurons.

### Ca_v_2.2/N‐type calcium channel trafficking is impaired in MAP6 KO neurons

Western blotting of protein extracts from various brain regions of WT and MAP6 KO animals, with an anti‐Ca_v_2.2/N‐type calcium channel antibody, did not reveal major expression deficits in this protein (Fig. 4A). Immunolabelling hippocampal neurons after 3–4 days in culture with an anti‐Ca_v_2.2/N‐type calcium channel antibody revealed the cell bodies as well as spots along neurites and growth cones (Fig. 4B). Global quantification of these signals did not reveal major expression deficits in the absence of MAP6, either. However, we reasoned that cell bodies being the initial localization of newly synthesized calcium channels and growth cones their final destination at this stage, measuring spots along neurites would better account for trafficking channels. Also, it has been suggested elsewhere that Tctex1 would interact with the cytoplasmic domain of parathyroid hormone‐related protein receptor and carry the molecule along the microtubules during the course of receptor internalization (Sugai *et al*., 2003). To measure the amount of Ca_v_2.2/N‐type calcium channels trafficking in MAP6 KO neurons, transfection of WT and MAP6 KO neurons with GFP before immunolabelling of calcium channels was realized and neuritic Ca_v_2.2/N‐type calcium channel content measured (Fig. 4C, see Supporting Information). Interestingly, these measurements revealed a 25% decrease in Ca_v_2.2/N‐type calcium channels in MAP6 KO vs. WT neurons (Fig. 4C, 0.758 ± 0.032 vs. 1.000 ± 0.041, *P *<* *0.001). In order to identify a more general deficit of trafficking, MAP6 KO and WT neurons were incubated with labelled transferrin (Tfn vesicles, see Material & Methods) to mark rapidly recycling endocytic vesicles on the one hand, and immunolabelled with an anti‐Lamp1 antibody to visualize mature lysosomes, on the other hand (Fig. 4D). Quantification of fluorescence intensities showed that MAP6 KO neurons displayed a small but significant deficit in Tfn‐labelled vesicles (Fig. 4D, 0.903 ± 0.007 vs. 1.000 ± 0.007, *P *<* *0.001) with no difference in Lamp1‐containing vesicles (Fig. 4D, 1.014 ± 0.007 vs. 1.000 ± 0.007, *P *=* *0.148) as compared to WT neurons. Hence, these observations point to impaired trafficking of Ca_v_2.2/N‐type calcium channels as a possible cause for diminished calcium signalling in MAP6 KO neurons. Decreased recycling, rather than increased lysosomal degradation, may be underlying this deficit.

### A Tctex1‐interacting MAP6 mutant is sufficient to restore trafficking of Ca_v_2.2/N‐type calcium channels

**Figure 5:**
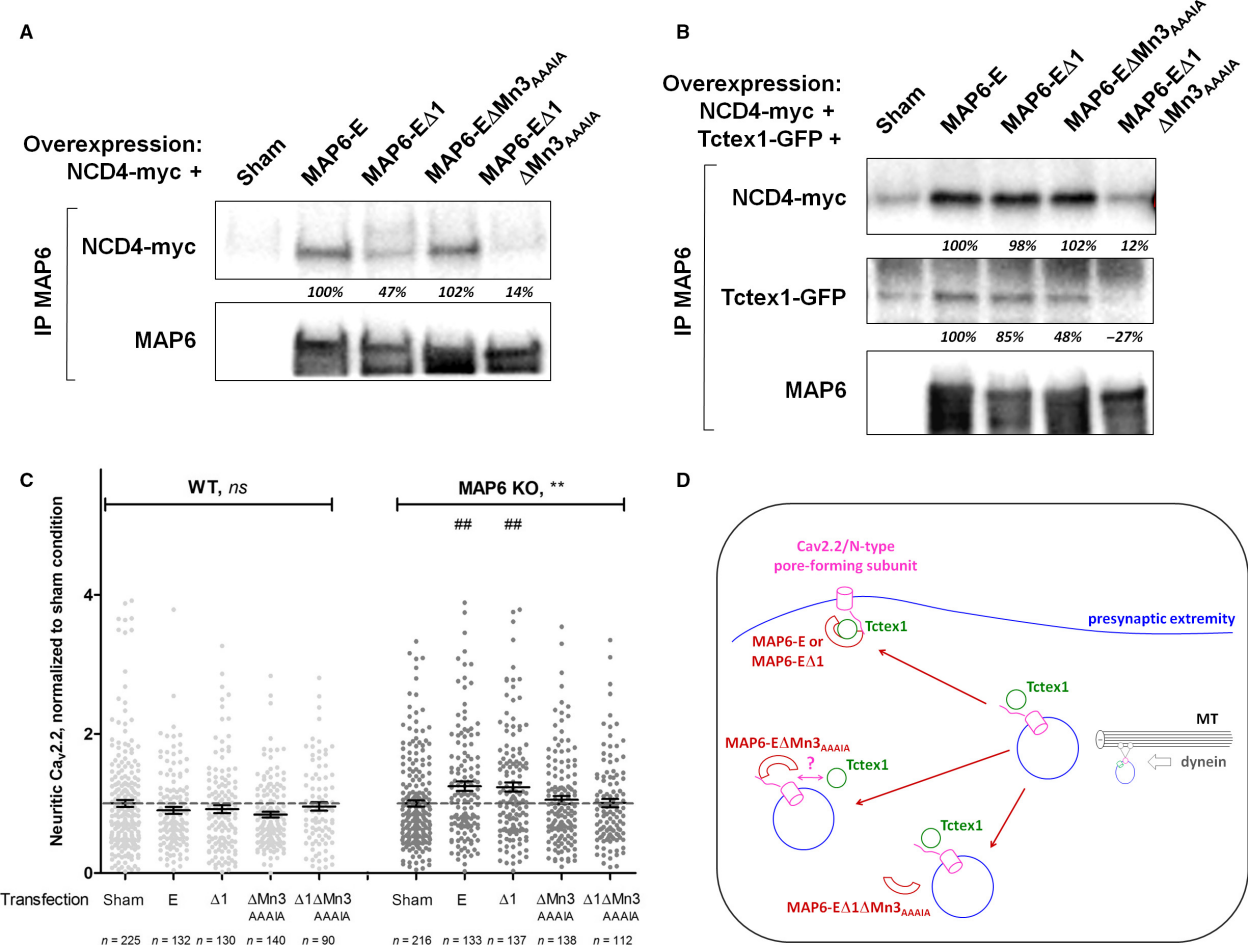
Interactions of MAP6‐E mutants with Ca_v_2.2/N‐type calcium channels and Tctex1, and restoration of neuritic content. (A) HEK‐293 T17 cells were cotransfected with plasmids encoding NCD4‐myc and full‐length MAP6‐E or mutants as in Fig. 1F. Each immunoprecipitation was performed with the anti‐MAP6 23N antibody and immunorevealed by Western blotting against myc or MAP6 (23N). Note that the many MAP6 bands detected after immunoprecipitation correspond to specific degradation products as there is no signal in the sham‐transfected condition. NCD4‐related bands were quantified, background subtracted and normalized by that obtained with non‐mutated MAP6‐E (italic percentages). (B) HEK‐293 T17 cells were cotransfected with plasmids encoding NCD4‐myc, Tctex1‐GFP and full‐length MAP6‐E or mutants as in Fig. 1F. Each immunoprecipitation was performed with the anti‐MAP6 23N antibody and immunorevealed by Western blotting against myc, GFP or MAP6 (23N). Note that the many MAP6 bands detected after immunoprecipitation correspond to specific degradation products as there is no signal in the sham‐transfected condition. NCD4‐related bands and Tctex1‐related bands were quantified, background subtracted and normalized by that obtained with non‐mutated MAP6‐E (italic percentages). (C) Quantification of neuritic Ca_v_2.2/N‐type calcium channels contents from WT (white discs) and MAP6 KO (grey discs) hippocampal neurons cotransfected with plasmids encoding GFP as in Fig. 4C and full‐length MAP6‐E or mutants as indicated, normalized by sham‐transfected neurons of corresponding genotype (dashed grey line = 100%). *n* represents the total number of transfected neurons measured from three independent neuronal cultures. ns, not significant and **, *P *<* *0.05, using a one‐way parametric anova, followed by Bonferroni's multiple comparison tests : ##, *P *<* *0.01 as compared to sham‐transfected KO. (D) Scheme of Ca_v_2.2/N‐type pore‐forming subunit being trafficked in presynaptic extremities from vesicles moving along microtubules. When vesicles are undocked, calcium channels are not addressed to the plasma membrane in the absence of MAP6 or in the presence of the mutant MAP6‐EΔ1ΔMn3_AAAIA_. In the presence of the mutant MAP6‐EΔMn3_AAAIA_, calcium channels are not properly routed either, although MAP6‐EΔMn3_AAAIA_ retains its ability to bind the channels. Functional calcium channels are restored only in the presence of both Tctex1 and MAP6‐E or mutant MAP6‐EΔ1. [Colour figure can be viewed at wileyonlinelibrary.com].

From previous results, it was not clear that MAP6 proteins would interact directly with calcium channels, in the absence of Tctex1. Hence, we attempted to co‐immunoprecipitate the C‐terminus of Ca_v_2.2/N‐type calcium channel [corresponding to the NCD4 construct (Lai *et al*., 2005)] with an anti‐MAP6 antibody (Fig. 5A). Full‐length MAP6‐E as well as the MAP6‐EΔMn3_AAAIA_ mutant was fully able to co‐immunoprecipitate NCD4. The MAP6‐EΔ1 mutant kept some affinity whereas the double‐mutant MAP6‐EΔ1ΔMn3_AAAIA_ lost all potential to co‐immunoprecipitate NCD4 (Fig. 5A). When confronted to both its partners in the same experiment, MAP6‐E and single mutants MAP6‐EΔ1 and MAP6‐EΔMn3_AAAIA_ behaved similarly, by co‐immunoprecipitating significant levels of both Tctex1 and NCD4. In contrast, the double‐mutant MAP6‐EΔ1ΔMn3_AAAIA_ showed no capacity to co‐immunoprecipitate either NCD4 or Tctex1 (Fig. 5B), thus confirming observations performed in the presence of each, separately (see Figs 1F and 5A). Finally, the capacity of the MAP6‐E mutants to restore Ca_v_2.2/N‐type channels trafficking was tested by overexpression in neurons, in addition to GFP used to visualize the neurites, as described earlier (Fig. 4C). As a control, WT neurons were transfected with plasmids encoding these mutants, but none modified Ca_v_2.2/N‐type channels trafficking significantly (Fig. 5C, *P* = 0.220). In MAP6 KO neurons however (Fig. 5C, *P* = 0.001), overexpression of MAP6‐E or MAP6‐EΔ1 significantly increased Ca_v_2.2/N‐type calcium channels trafficking (1.235 ± 0.067 and 1.248 ± 0.069, respectively, as compared to 1.000 ± 0.044 for sham‐transfected condition, *P *<* *0.01 for each) when neither MAP6‐EΔMn3_AAAIA_ nor MAP6‐EΔ1ΔMn3_AAAIA_ showed such activity (1.054 ± 0.055 and 1.005 ± 0.059, respectively). Interestingly, the levels of neuritic Ca_v_2.2/N‐type calcium channels measured in WT or in MAP6 KO neurons overexpressing MAP6‐E or MAP6‐EΔ1 no longer differed (Fig. S3).

## Discussion

Tctex1 was initially identified as a facultative light chain of brain cytoplasmic dynein (King *et al*., 1996), used for the apical transport of specific cargoes (Tai *et al*., 2001). Since, it has been described as a bona fide interaction partner in a number of yeast two‐hybrid screenings, for example that of the full‐length C‐terminus of the Ca_v_2.2/N‐type pore‐forming subunit (Lai *et al*., 2005). In our hands, Tctex1 was also identified from a yeast two‐hybrid screening, using the C‐terminus of MAP6‐N as bait. This interaction was confirmed with both neuronal isoforms, MAP6‐E and MAP6‐N, using co‐immunoprecipitation experiments. In concordance, minimal interaction sequences of MAP6 have been identified in a segment common to the two isoforms (aa452–572, see Fig. 1). A putative consensus interaction sequence R/X‐R/X‐R/X‐X‐R/X with Tctex1 was detected at aa532–536 of the MAP6 sequence. When this sequence was mutated to AAAIA however, the binding to Tctex1 was not diminished significantly. Note that other groups have shown that the presence of the consensus signal is not obligatory for a protein of interest to display Tctex1 binding (Ochiai *et al*., 2011). Other sequences involved in the interaction with Tctex1 were identified thanks to sequence homologies between MAP6 proteins and its homologue MAP6d1 (Bosc *et al*., 2003). Indeed, the N‐terminus (Gory‐Faure *et al*., 2006) and a microtubule‐binding domain called Mn3 (Bosc *et al*., 2001) are shared by MAP6 proteins and MAP6d1 and are both involved in the interaction with Tctex1. Nevertheless, maintained expression of MAP6d1 in MAP6 KO neurons did not seem to compensate for the absence of MAP6 proteins, responsible for the decreased calcium signalling in this model, as discussed below.

As Tctex1 was shown to participate in the surface expression of specific calcium channels, we tested the integrity of calcium signalling in MAP6 KO neurons. Using electrophysiology recordings and calcium imaging, we demonstrated that calcium influxes supported by Ca_v_2‐type calcium channels were diminished in MAP6 KO as compared to WT neurons. The amplitude of the decrease varied from one technique to another, probably due to the specific cocktail of inhibitors used for each. Besides, calcium imaging revealed decreased calcium signalling more readily in the neurites than in the cell bodies of MAP6 KO neurons. Addition of kynurenic acid, an inhibitor of NMDA receptors, to the imaging medium was necessary to detect such a decrease in the cell bodies as well. This indicated that NMDA receptors may be slightly overexpressed in cell bodies of MAP6 KO neurons as compared to WT, an observation that may be related to global deficient neuritic transport. Alternatively, NMDA receptors' sensitivity to glutamate may be increased in MAP6 KO neurons in adaptation to the global brain hypoglutamatergy reported in MAP6 KO mice (Brenner *et al*., 2007).

Amongst Ca_v_2‐type calcium channels, the different pore‐forming subunits are structurally homologous but not identical; hence, they can be distinctly inhibited by very specific toxins, purified from cone snails' or spiders' venom: ω‐agatoxin IVA against Ca_v_2.1/PQ‐type, ω‐conotoxin GVIA against Ca_v_2.2/N‐type and SNX‐482 against Ca_v_2.3/R‐type (Catterall *et al*., 2005). Using cortical cultures, we established that Ca_v_2.2/N‐type channels were functionally impaired in the absence of MAP6, thereby evoking similar results obtained in the absence of Tctex1 (Lai *et al*., 2005). We also tested axonal polarization as a biological phenomenon supported by Ca_v_2.2/N‐type calcium channels mostly (Pravettoni *et al*., 2000) and showed that, in contrast with WT neurons, MAP6 KO neurons were impervious to the presence of ω‐conotoxin in the culture medium. Also, mature neuronal networks were tested for spontaneous calcium activity, a biological feature that heavily depends on Ca_v_2.1/PQ‐type calcium channels (Qian & Noebels, 2001). Spontaneous activity was slightly less intense in MAP6 KO neurons as compared to WT neurons and significantly so in neurites only. This would reinforce the observation that functional Ca_v_2‐type calcium channels are possibly missing in presynaptic boutons, in the absence of MAP6. Alternatively, intracellular stores may be affected in MAP6 KO neurons although mitochondrial capacity to buffer calcium seemed intact in preliminary experiments (not shown). Note that no experiments have been performed towards documenting diminished Ca_v_2.3/R‐type calcium channels in MAP6 KO neurons as this type is already quite minor in hippocampal neurons (Schlick *et al*., 2010). Interestingly, the possibility that MAP6 proteins may regulate every member of the Ca_v_2‐type family echoes with previous proteomics results showing that MAP6 proteins are found in similar abundance in Ca_v_2.1/PQ‐type, Ca_v_2.2/N‐type and Ca_v_2.3/R‐type calcium channels nanoenvironments (Muller *et al*., 2010).

We next tried to establish a cause for missing functional calcium channels in MAP6 KO neurons, bearing in mind that a similar phenotype was obtained when Tctex1 interactions with Ca_v_2.2/N‐type calcium channels were inhibited (Lai *et al*., 2005). In the present study, the total expression of Ca_v_2.2/N‐type channels was unchanged in various brain regions from MAP6 KO mice as compared to WT. Similarly, immunolabelling Ca_v_2.2/N‐type channels in MAP6 KO hippocampal neurons did not reveal any global decrease in expression. It thus appeared that in the absence of one or the other member of the MAP6‐Tctex1 pair, Ca_v_2.2/N‐type calcium channel total expression was intact whereas its surface expression was altered. Interestingly, when measuring Ca_v_2.2/N‐type calcium channels immunodetected within neurites of transfected neurons only, it was possible to detect diminished neuritic content, in accordance with a possible lesser transport. We also estimated the recycling and degradation capacities of MAP6 KO neurons via immunolabelling of Tfn‐positive and Lamp1‐positive vesicles, respectively. We demonstrated that the short recycling loop was more likely to be impaired in the absence of MAP6, than the lysosomal degradation to be increased. Thus, more probably than routing newly expressed channels, the Tctex1‐MAP6 pair may be modulating their recycling in hippocampal neurons, at later stages. This hypothesis would by in accordance with the fact that inhibiting Tctex1's interactions with Ca_v_2.2/N‐type channels needed more than 48 h to offer a measurable decrease in electrophysiological recordings (Lai *et al*., 2005).

Lastly, we showed via complementation experiments that MAP6‐E was enough to restore calcium signalling and neuritic Ca_v_2.2/N‐type content when overexpressed in MAP6 KO neurons. Interestingly, a similar overexpression initiated no such modifications in WT neurons. This could be due to the limitation in the number of ‘slots’ available to each type of calcium channels in the presynaptic compartment of cultured neurons (Cao & Tsien, 2010) or to the limiting amount of chaperone proteins involved in the accurate location of calcium channels (Hoppa *et al*., 2012). It could also be indicating that the balance of partners to establish functional Tctex1‐MAP6 pairs is important in regulating calcium channel trafficking. This last hypothesis seems in line with our final immunoprecipitation results obtained with MAP6‐EΔ1 and MAP6‐EΔMn3_AAAIA_: when the latter seems to strongly bind NCD4 but less so Tctex1, it does not restore calcium channels trafficking, whereas the former keeps a strong interaction with Tctex1, much less vis‐à‐vis NCD4, but does restore trafficking of Ca_v_2.2/N‐type channels effectively (Fig. 5D). In any case, only the mutation of both regions prevents interaction with both partners as well as restoration of functional calcium channels.

In conclusion, we demonstrate in the present study that MAP6 proteins interact with Tctex1 and Ca_v_2.2/N‐type calcium channel to regulate calcium channel activity in hippocampal neurons. Interestingly, this is yet additional evidence showing that MAP6 is involved in vesicular trafficking in neurons (Schwenk *et al*., 2014; Tortosa *et al*., 2017).

## Conflict of interest

The authors declare no conflict of interest.

## Author contributions

JB, FD, CA, CB, ED, LP, YS and SG contributed to the experiments; JB, MDW, SG and AA contributed to design the study; JB, CA, ED and SG contributed to analyse the data; and JB, CA, CB, ED, MDW, SG and AA contributed to the preparation of the manuscript.

## Data accessibility

All primary data generated in this study have been made freely available via Figshare.

## Supporting information

Fig. S1. Mapping of Tctex1 binding site on MAP6‐N peptide array.
Fig. S2. MAP6d1 interacts with Tctex1.
Fig. S3. MAP6‐E or MAP6‐EΔ1 overexpression restores the levels of neuritic Ca_v_2.2 in MAP6 KO neurons.

## References

[ejn13766-bib-0001] Andrieux, A. , Salin, P.A. , Vernet, M. , Kujala, P. , Baratier, J. , Gory‐Faure, S. , Bosc, C. , Pointu, H. *et al* (2002) The suppression of brain cold‐stable microtubules in mice induces synaptic defects associated with neuroleptic‐sensitive behavioral disorders. Genes Dev., 16, 2350–2364. 12231625 10.1101/gad.223302 PMC187434

[ejn13766-bib-0002] Baratier, J. , Peris, L. , Brocard, J. , Gory‐Faure, S. , Dufour, F. , Bosc, C. , Fourest‐Lieuvin, A. , Blanchoin, L. *et al* (2006) Phosphorylation of microtubule‐associated protein STOP by calmodulin kinase II. J. Biol. Chem., 281, 19561–19569. 16651267 10.1074/jbc.M509602200

[ejn13766-bib-0003] Bosc, C. , Frank, R. , Denarier, E. , Ronjat, M. , Schweitzer, A. , Wehland, J. & Job, D. (2001) Identification of novel bifunctional calmodulin‐binding and microtubule‐stabilizing motifs in STOP proteins. J. Biol. Chem., 276, 30904–30913. 11413126 10.1074/jbc.M011614200

[ejn13766-bib-0004] Bosc, C. , Andrieux, A. & Job, D. (2003) STOP proteins. Biochemistry, 42, 12125–12132. 14567673 10.1021/bi0352163

[ejn13766-bib-0005] Brenner, E. , Sonnewald, U. , Schweitzer, A. , Andrieux, A. & Nehlig, A. (2007) Hypoglutamatergic activity in the STOP knockout mouse: a potential model for chronic untreated schizophrenia. J. Neurosci. Res., 85, 3487–3493. 17304567 10.1002/jnr.21200

[ejn13766-bib-0006] Brun, P. , Begou, M. , Andrieux, A. , Mouly‐Badina, L. , Clerget, M. , Schweitzer, A. , Scarna, H. , Renaud, B. *et al* (2005) Dopaminergic transmission in STOP null mice. J. Neurochem., 94, 63–73. 15953350 10.1111/j.1471-4159.2005.03166.x

[ejn13766-bib-0007] Cao, Y.Q. & Tsien, R.W. (2010) Different relationship of N‐ and P/Q‐type Ca2 + channels to channel‐interacting slots in controlling neurotransmission at cultured hippocampal synapses. J. Neurosci., 30, 4536–4546. 20357104 10.1523/JNEUROSCI.5161-09.2010 PMC3842455

[ejn13766-bib-0008] Catterall, W.A. , Perez‐Reyes, E. , Snutch, T.P. & Striessnig, J. (2005) International Union of Pharmacology. XLVIII. Nomenclature and structure‐function relationships of voltage‐gated calcium channels. Pharmacol. Rev., 57, 411–425. 16382099 10.1124/pr.57.4.5

[ejn13766-bib-0009] Chuang, J.Z. , Yeh, T.Y. , Bollati, F. , Conde, C. , Canavosio, F. , Caceres, A. & Sung, C.H. (2005) The dynein light chain Tctex‐1 has a dynein‐independent role in actin remodeling during neurite outgrowth. Dev. Cell, 9, 75–86. 15992542 10.1016/j.devcel.2005.04.003 PMC3857739

[ejn13766-bib-0010] Deloulme, J.C. , Gory‐Faure, S. , Mauconduit, F. , Chauvet, S. , Jonckheere, J. , Boulan, B. , Mire, E. , Xue, J. *et al* (2015) Microtubule‐associated protein 6 mediates neuronal connectivity through Semaphorin 3E‐dependent signalling for axonal growth. Nat. Commun., 6, 7246. 26037503 10.1038/ncomms8246 PMC4468860

[ejn13766-bib-0011] Delphin, C. , Bouvier, D. , Seggio, M. , Couriol, E. , Saoudi, Y. , Denarier, E. , Bosc, C. , Valiron, O. *et al* (2012) MAP6‐F is a temperature sensor that directly binds to and protects microtubules from cold‐induced depolymerization. J. Biol. Chem., 287, 35127–35138. 22904321 10.1074/jbc.M112.398339 PMC3471743

[ejn13766-bib-0012] Denarier, E. , Aguezzoul, M. , Jolly, C. , Vourc'h, C. , Roure, A. , Andrieux, A. , Bosc, C. & Job, D. (1998a) Genomic structure and chromosomal mapping of the mouse STOP gene (Mtap6). Biochem. Bioph. Res. Co., 243, 791–796. 10.1006/bbrc.1998.8179 9501006

[ejn13766-bib-0013] Denarier, E. , Fourest‐Lieuvin, A. , Bosc, C. , Pirollet, F. , Chapel, A. , Margolis, R.L. & Job, D. (1998b) Nonneuronal isoforms of STOP protein are responsible for microtubule cold stability in mammalian fibroblasts. Proc. Natl. Acad. Sci. USA, 95, 6055–6060. 9600916 10.1073/pnas.95.11.6055 PMC27584

[ejn13766-bib-0014] Dolphin, A.C. (2016) Voltage‐gated calcium channels and their auxiliary subunits: physiology and pathophysiology and pharmacology. J. Physiol., 594, 5369–5390. 27273705 10.1113/JP272262 PMC5043047

[ejn13766-bib-0015] Douglas, M.W. , Diefenbach, R.J. , Homa, F.L. , Miranda‐Saksena, M. , Rixon, F.J. , Vittone, V. , Byth, K. & Cunningham, A.L. (2004) Herpes simplex virus type 1 capsid protein VP26 interacts with dynein light chains RP3 and Tctex1 and plays a role in retrograde cellular transport. J. Biol. Chem., 279, 28522–28530. 15117959 10.1074/jbc.M311671200

[ejn13766-bib-0016] Fournet, V. , Jany, M. , Fabre, V. , Chali, F. , Orsal, D. , Schweitzer, A. , Andrieux, A. , Messanvi, F. *et al* (2010) The deletion of the microtubule‐associated STOP protein affects the serotonergic mouse brain network. J. Neurochem., 115, 1579–1594. 20969568 10.1111/j.1471-4159.2010.07064.x

[ejn13766-bib-0017] Gauthier‐Fisher, A. , Lin, D.C. , Greeve, M. , Kaplan, D.R. , Rottapel, R. & Miller, F.D. (2009) Lfc and Tctex‐1 regulate the genesis of neurons from cortical precursor cells. Nat. Neurosci., 12, 735–744. 19448628 10.1038/nn.2339

[ejn13766-bib-0018] Gory‐Faure, S. , Windscheid, V. , Bosc, C. , Peris, L. , Proietto, D. , Franck, R. , Denarier, E. , Job, D. *et al* (2006) STOP‐like protein 21 is a novel member of the STOP family, revealing a Golgi localization of STOP proteins. J. Biol. Chem., 281, 28387–28396. 16837464 10.1074/jbc.M603380200

[ejn13766-bib-0019] Gory‐Faure, S. , Windscheid, V. , Brocard, J. , Montessuit, S. , Tsutsumi, R. , Denarier, E. , Fukata, Y. , Bosc, C. *et al* (2014) Non‐Microtubular Localizations of Microtubule‐Associated Protein 6 (MAP6). PLoS One, 9, e114905. 25526643 10.1371/journal.pone.0114905 PMC4272302

[ejn13766-bib-0020] Guillaud, L. , Bosc, C. , Fourest‐Lieuvin, A. , Denarier, E. , Pirollet, F. , Lafanechere, L. & Job, D. (1998) STOP proteins are responsible for the high degree of microtubule stabilization observed in neuronal cells. J. Cell Biol., 142, 167–179. 9660871 10.1083/jcb.142.1.167 PMC2133033

[ejn13766-bib-0021] Hoppa, M.B. , Lana, B. , Margas, W. , Dolphin, A.C. & Ryan, T.A. (2012) alpha2delta expression sets presynaptic calcium channel abundance and release probability. Nature, 486, 122–125. 22678293 10.1038/nature11033 PMC3376018

[ejn13766-bib-0022] Khodorov, B. , Pinelis, V. , Vinskaya, N. , Sorokina, E. , Grigortsevich, N. & Storozhevykh, T. (1999) Li+ protects nerve cells against destabilization of Ca2 + homeostasis and delayed death caused by removal of external Na+. FEBS Lett., 448, 173–176. 10217434 10.1016/s0014-5793(99)00350-6

[ejn13766-bib-0023] King, S.M. , Dillman, J.F. 3rd , Benashski, S.E. , Lye, R.J. , Patel‐King, R.S. & Pfister, K.K. (1996) The mouse t‐complex‐encoded protein Tctex‐1 is a light chain of brain cytoplasmic dynein. J. Biol. Chem., 271, 32281–32287. 8943288 10.1074/jbc.271.50.32281

[ejn13766-bib-0024] Kingston, R.E. , Chen, C.A. & Okayama, H. (2001) Calcium phosphate transfection. Curr. Protoc. Neurosci., **Appendix 1**, Appendix 1C. 10.1002/0471142301.nsa01cs01 18428434

[ejn13766-bib-0025] Lai, M. , Wang, F. , Rohan, J.G. , Maeno‐Hikichi, Y. , Chen, Y. , Zhou, Y. , Gao, G. , Sather, W.A. *et al* (2005) A tctex1‐Ca2 + channel complex for selective surface expression of Ca2 + channels in neurons. Nat. Neurosci., 8, 435–442. 15768038 10.1038/nn1418

[ejn13766-bib-0026] Lee, A. , Scheuer, T. & Catterall, W.A. (2000) Ca2 + /calmodulin‐dependent facilitation and inactivation of P/Q‐type Ca2 + channels. J. Neurosci., 20, 6830–6838. 10995827 10.1523/JNEUROSCI.20-18-06830.2000 PMC6772829

[ejn13766-bib-0027] Margolis, R.L. , Rauch, C.T. & Job, D. (1986) Purification and assay of a 145‐kDa protein (STOP145) with microtubule‐stabilizing and motility behavior. Proc. Natl. Acad. Sci. USA, 83, 639–643. 3456161 10.1073/pnas.83.3.639 PMC322919

[ejn13766-bib-0028] Maximov, A. , Sudhof, T.C. & Bezprozvanny, I. (1999) Association of neuronal calcium channels with modular adaptor proteins. J. Biol. Chem., 274, 24453–24456. 10455105 10.1074/jbc.274.35.24453

[ejn13766-bib-0029] Miller, R.J. (1987) Multiple calcium channels and neuronal function. Science, 235, 46–52. 2432656 10.1126/science.2432656

[ejn13766-bib-0030] Mok, Y.K. , Lo, K.W. & Zhang, M. (2001) Structure of Tctex‐1 and its interaction with cytoplasmic dynein intermediate chain. J. Biol. Chem., 276, 14067–14074. 11148215 10.1074/jbc.M011358200

[ejn13766-bib-0031] Muller, C.S. , Haupt, A. , Bildl, W. , Schindler, J. , Knaus, H.G. , Meissner, M. , Rammner, B. , Striessnig, J. *et al* (2010) Quantitative proteomics of the Cav2 channel nano‐environments in the mammalian brain. Proc. Natl. Acad. Sci. USA, 107, 14950–14957. 20668236 10.1073/pnas.1005940107 PMC2930569

[ejn13766-bib-0032] Ochiai, K. , Watanabe, M. , Ueki, H. , Huang, P. , Fujii, Y. , Nasu, Y. , Noguchi, H. , Hirata, T. *et al* (2011) Tumor suppressor REIC/Dkk‐3 interacts with the dynein light chain, Tctex‐1. Biochem. Bioph. Res. Co., 412, 391–395. 10.1016/j.bbrc.2011.07.109 21835165

[ejn13766-bib-0033] Paturle‐Lafanechere, L. , Manier, M. , Trigault, N. , Pirollet, F. , Mazarguil, H. & Job, D. (1994) Accumulation of delta 2‐tubulin, a major tubulin variant that cannot be tyrosinated, in neuronal tissues and in stable microtubule assemblies. J. Cell Sci., 107(Pt 6), 1529–1543. 7962195 10.1242/jcs.107.6.1529

[ejn13766-bib-0034] Pirollet, F. , Rauch, C.T. , Job, D. & Margolis, R.L. (1989) Monoclonal antibody to microtubule‐associated STOP protein: affinity purification of neuronal STOP activity and comparison of antigen with activity in neuronal and nonneuronal cell extracts. Biochemistry, 28, 835–842. 2713350 10.1021/bi00428a064

[ejn13766-bib-0035] Pravettoni, E. , Bacci, A. , Coco, S. , Forbicini, P. , Matteoli, M. & Verderio, C. (2000) Different localizations and functions of L‐type and N‐type calcium channels during development of hippocampal neurons. Dev. Biol., 227, 581–594. 11071776 10.1006/dbio.2000.9872

[ejn13766-bib-0036] Qian, J. & Noebels, J.L. (2001) Presynaptic Ca2 + channels and neurotransmitter release at the terminal of a mouse cortical neuron. J. Neurosci., 21, 3721–3728. 11356859 10.1523/JNEUROSCI.21-11-03721.2001 PMC6762720

[ejn13766-bib-0037] Schlick, B. , Flucher, B.E. & Obermair, G.J. (2010) Voltage‐activated calcium channel expression profiles in mouse brain and cultured hippocampal neurons. Neuroscience, 167, 786–798. 20188150 10.1016/j.neuroscience.2010.02.037 PMC3315124

[ejn13766-bib-0038] Schneider, C.A. , Rasband, W.S. & Eliceiri, K.W. (2012) NIH Image to ImageJ: 25 years of image analysis. Nat. Methods, 9, 671–675. 22930834 10.1038/nmeth.2089 PMC5554542

[ejn13766-bib-0039] Schwenk, B.M. , Lang, C.M. , Hogl, S. , Tahirovic, S. , Orozco, D. , Rentzsch, K. , Lichtenthaler, S.F. , Hoogenraad, C.C. *et al* (2014) The FTLD risk factor TMEM106B and MAP6 control dendritic trafficking of lysosomes. EMBO J., 33, 450–467. 24357581 10.1002/embj.201385857 PMC3989627

[ejn13766-bib-0040] Sugai, M. , Saito, M. , Sukegawa, I. , Katsushima, Y. , Kinouchi, Y. , Nakahata, N. , Shimosegawa, T. , Yanagisawa, T. *et al* (2003) PTH/PTH‐related protein receptor interacts directly with Tctex‐1 through its COOH terminus. Biochem. Bioph. Res. Co., 311, 24–31. 10.1016/j.bbrc.2003.09.157 14575690

[ejn13766-bib-0041] Tai, A.W. , Chuang, J.Z. & Sung, C.H. (2001) Cytoplasmic dynein regulation by subunit heterogeneity and its role in apical transport. J. Cell Biol., 153, 1499–1509. 11425878 10.1083/jcb.153.7.1499 PMC2150720

[ejn13766-bib-0042] Tortosa, E. , Adolfs, Y. , Fukata, M. , Pasterkamp, R.J. , Kapitein, L.C. & Hoogenraad, C.C. (2017) Dynamic Palmitoylation Targets MAP6 to the Axon to Promote Microtubule Stabilization during Neuronal Polarization. Neuron, 94, 809–825. e807. 28521134 10.1016/j.neuron.2017.04.042

[ejn13766-bib-0043] Wehland, J. & Willingham, M.C. (1983) A rat monoclonal antibody tpdel reacting specifically with the tyrosylated form of alpha‐tubulin. II. Effects on cell movement, organization of microtubules, and intermediate filaments, and arrangement of Golgi elements. J. Cell Biol., 97, 1476–1490. 6685128 10.1083/jcb.97.5.1476 PMC2112707

