# Supplementary material for: MAP6 interacts with Tctex1 and Cav2.2/N‐type calcium channels to regulate calcium signalling in neurons
Source: Eur J Neurosci. 2017 Nov 22;46(11):2754–67. doi: 10.1111/ejn.13766 (PMC5765474; doi:10.1111/ejn.13766)
Supplement: Supplementary file 1 — Fig. S1. Mapping of Tctex1 binding site on MAP6‐N peptide array. Fig. S2. MAP6d1 interacts with Tctex1. Fig. S3. MAP6‐E or MAP6‐EΔ1 overexpression restores the levels of neuritic Cav2.2 in MAP6 KO neurons. [file EJN-46-2754-s001.docx]

**Supplemental Material**


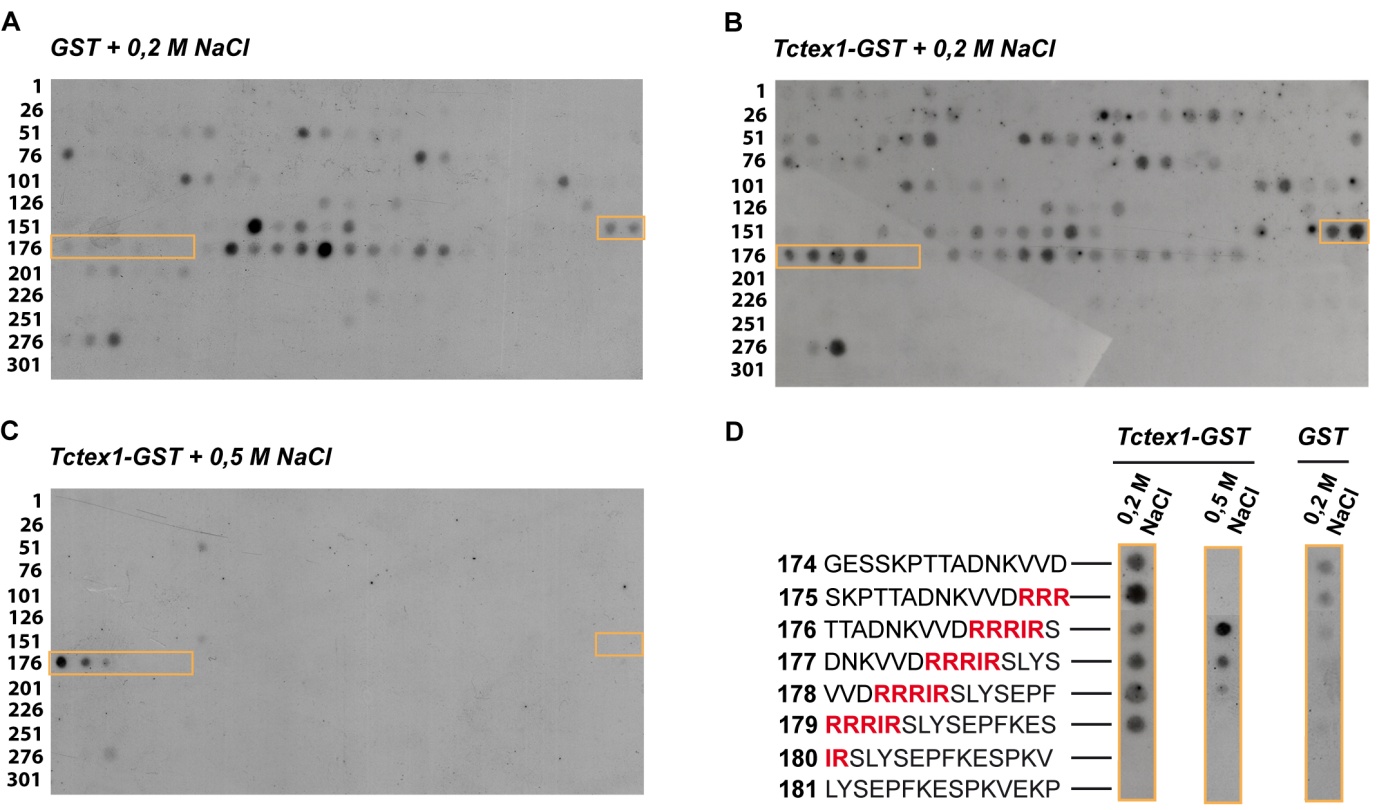


**Figure S1 - Mapping of Tctex1 binding site on MAP6-N peptide array**

(A-C) Overlays of GST (A) or Tctex1-GST (B-C) on membranes containing immobilized MAP6N 15-aa overlapping peptide arrays. The last 12 aa of each peptide overlap the first 12 aa of the next peptide (see examples in D). Peptides were numbered from the amino-terminal to the carboxyl-terminal residues of MAP6-N and numbers correspond to the first peptide of each line. Binding assays were performed in the presence of either 0.2 M NaCl (A-B) or 0.5 M NaCl (C). Bound GST or Tctex1-GSP proteins were immunodetected using an anti-GST antibody. A cluster of amino acids potentially specific for the interaction was framed in orange (see below).

(D) Immunodetection intensity for a cluster of peptides (174 to 181) detailed from results shown in A-C (orange frames), with the corresponding peptide sequences. Red : MAP6 aa coresponding to the Tctex1-binding consensus sequence R/K-R/K-X-X-R/K.

**
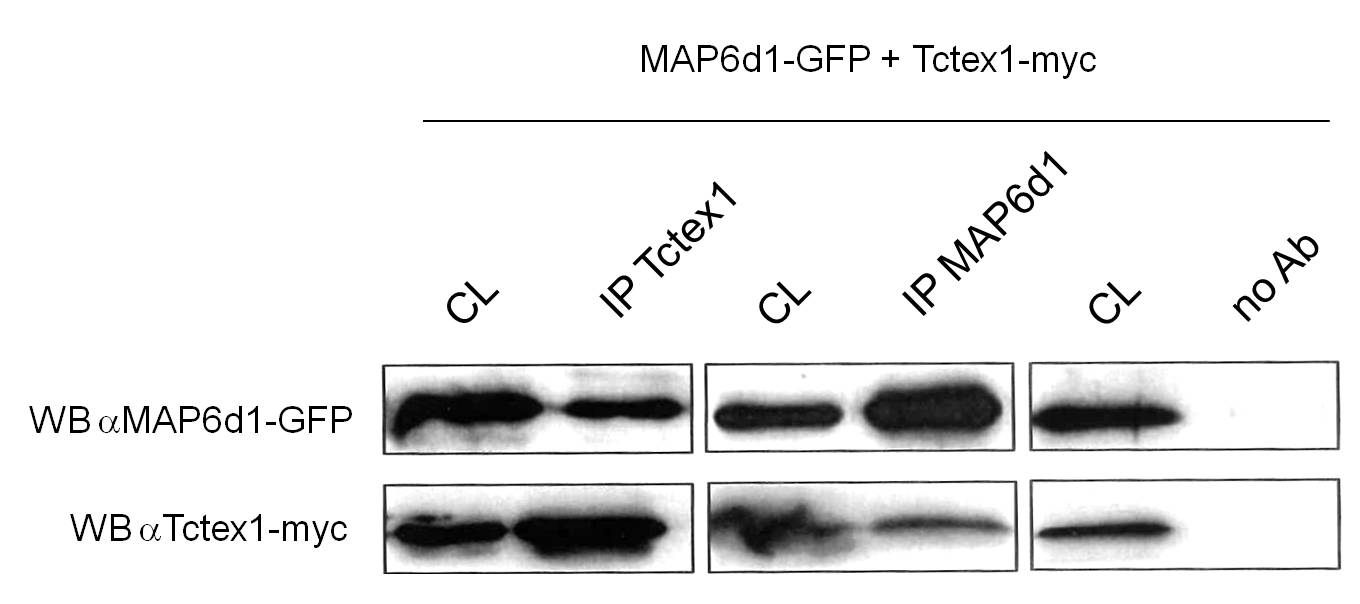
**

**Figure S2 - MAP6d1 interacts with Tctex1**

Immunoprecipitation of protein complexes obtained from COS-7 cells transfected with plasmids encoding Tctex1-myc and MAP6d1-GFP (Gory-Faure *et al.*, 2006), using sepharose beads coupled to an anti-myc (IP Tctex1) or an anti-GFP (IP MAP6d1) antibody. Cell lysates (CL) and immunoprecipitated protein complexes (IP) were then analyzed by SDS-PAGE and Western Blotting, using an anti-GFP (to evidence MAP6d1) or an anti-myc (to evidence Tctex1) antibody. Control experiments were performed with no antibody coupled to the beads (no Ab).

**Reference**

Gory-Faure, S., Windscheid, V., Bosc, C., Peris, L., Proietto, D., Franck, R., Denarier, E., Job, D. & Andrieux, A. (2006) STOP-like protein 21 is a novel member of the STOP family, revealing a Golgi localization of STOP proteins. *J Biol Chem*, **281**, 28387-28396.


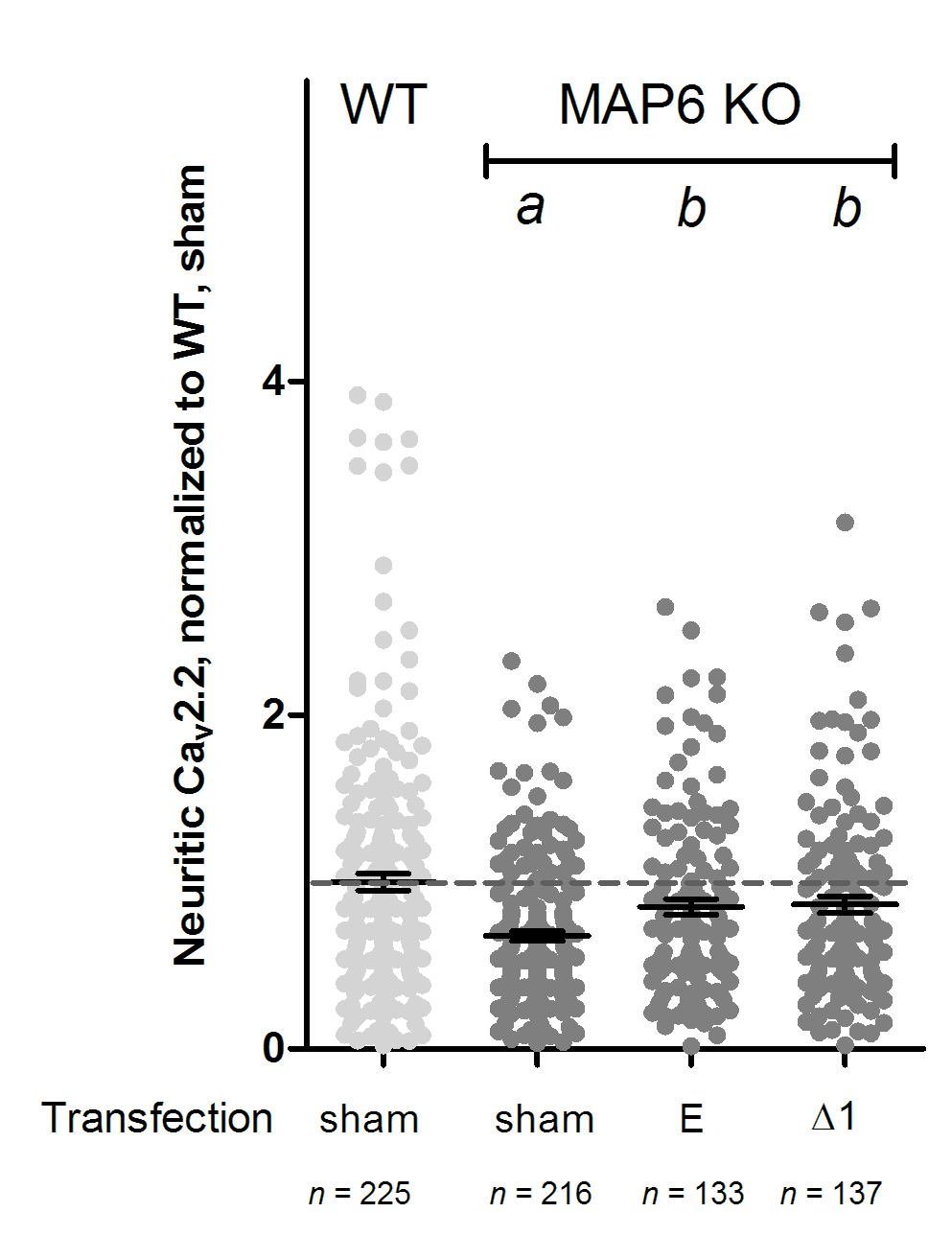


**Figure S3 - MAP6-E or MAP6-EΔ1 overexpression restores the levels of neuritic Ca_v_2.2 in MAP6 KO neurons**

Quantification of neuritic Ca_v_2.2/N-type calcium channels contents from WT (white disks) and MAP6 KO (grey disks) hippocampal neurons co-transfected with plasmids encoding GFP and full length MAP6-E or mutants as indicated, normalized by sham-transfected WT neurons (dashed grey line = 100%). *n* represents the total number of transfected neurons measured from 3 independent neuronal cultures. ***, *p* < 0.001, using a one-way parametric ANOVA, followed by Bonferroni's Multiple Comparison Tests to define significantly different groups: (*a*) *p* < 0.001 as compared to WT, sham and (*b*) *p* < 0.05 as compared to KO, sham and non-significant as compared to WT, sham.

**Production of an anti-Tctex1 antibody**

Fig. 1, see **Immunoprecipitations**. Polyclonal antibodies have been obtained by injecting two rabbits with a mix of two independent mouse Tctex1 peptides (EDFQASEETAF and VSSIVKEAIESAIGG, respectively corresponding to aa 2-12 and 17-31, according to accession number NP_033368), with an acetylation of the amino-group to mimic an internal peptide bond and an additional C-terminal cysteine residue for the coupling to Keyhole Limpet Hemocyanin (Eurogentec). Sera were affinity-purified against the peptides EDFQASEETAF or VSSIVKEAIESAIGG to generate antibodies 2025 and 2026, respectively.

**Image Analysis**

Fig. 2, see **Calcium Imaging**. For each time-lapse movie, regions of interest for cell bodies and neurites were detected with ImageJ from a projection of every image (see Macro1 below) and fluorescence intensity variations were measured in these regions. Baseline-subtracted peak intensity obtained during the second stimulation - in the presence of nimodipine - was divided by baseline-subtracted peak intensity obtained during the first stimulation, with KCl alone using Microsoft Excel^®^ software. When neurons were transfected prior to calcium imaging (Fig. 2E), an additional micrograph of the transfected neuron labeled in red was used to detect the cell body and neurites of the transfected neuron, specifically (see Macro2 below).

Fig. 3, see **Spontaneous activity**. For each time-lapse movie, regions of interest for cell bodies and neurites were detected with ImageJ from a projection of every image (see Macro1 below) and fluorescence intensity variations were measured in these regions. Signal analysis was performed using Microsoft Excel^®^ software: peak detection was triggered for each increase larger than 1% of the baseline between two time points and lasted until the signal decreased. Peaks were validated when their intensity was greater than 2% of the baseline.

Fig. 4, see **Immunolabelings**. (B) For each micrograph, Cav2.2 spots were detected with ImageJ (see Macro3 below) and logarithms of corresponding areas were averaged from the 5-20 images of each neuronal culture. For each of 4 independent culture, the average obtained for each neuronal culture was normalized by the mean value of Cav2.2 spots area of WT embryos measured that day. (C) For each micrograph, a region of interest was manually drawn around GFP-labeled neurites and skeletonization as well as Cav2.2 spots detection was performed using ImageJ (see Macro4 below). Neuritic Cav2.2 contents were measured for each transfected neuron and normalized by the mean value of neuritic Cav2.2 contents detected in the WT condition.

Fig. 5, see **Immunolabelings** (C) For each micrograph, a region of interest was manually drawn around GFP-labeled neurites and skeletonization as well as Cav2.2 spots detection was performed using ImageJ (see Macro4 below). Neuritic Cav2.2 contents were measured for each transfected neuron and normalized by the mean value of neuritic Cav2.2 contents detected in the WT, sham or MAP6 KO, sham conditions.

Macro1

rename("Stack");

run("Z Project...", "start=2 stop=200 projection=[Max Intensity]");

run("Select All");

run("Copy");

run("Subtract Background...", "rolling=50");

run("Internal Clipboard");

run("Gaussian Blur...", "sigma=1");

run("Subtract Background...", "rolling=50");

**Manual thresholding of both images**

run("Options...", "iterations=4 count=1 black edm=Overwrite do=Nothing");

setOption("BlackBackground", true);

selectWindow("Clipboard");

run("Dilate");

imageCalculator("Subtract create", "MAX_Stack","Clipboard");

selectWindow("Result of MAX_Stack");

run("Watershed");

run("Analyze Particles...", "size=10-Infinity circularity=0.00-1.00 show=Nothing add");

roiManager("Show All with labels");

roiManager("Show All");

roiManager("Combine");

run("Close");

run("Add to Manager");

Macro2

rename("Stack");

run("Select All");

run("Copy");

run("Internal Clipboard");

run("Subtract Background...", "rolling=50");

selectWindow("Stack");

run("Z Project...", "start=2 stop=200 projection=[Max Intensity]");

run("Select All");

run("Copy");

run("Internal Clipboard");

run("Subtract Background...", "rolling=50");

run("Internal Clipboard");

run("Gaussian Blur...", "sigma=1");

run("Subtract Background...", "rolling=50");

selectWindow("MAX_Stack");

close();

**Manual thresholding of the three images**

run("Options...", "iterations=4 count=1 black edm=Overwrite do=Nothing");

setOption("BlackBackground", true);

selectWindow("Clipboard-2");

run("Dilate");

imageCalculator("Subtract create", "Clipboard","Clipboard-2");

selectWindow("Result of Clipboard");

run("Watershed");

run("Analyze Particles...", "size=10-Infinity circularity=0.00-1.00 show=Nothing add");

roiManager("Show All with labels");

roiManager("Show All");

roiManager("Combine");

run("Close");

run("Add to Manager");

**Save the defined region of interest corresponding to the transfected neuron**

selectWindow("Clipboard");

setOption("BlackBackground", true);

run("Dilate");

imageCalculator("Subtract create", "Clipboard-1","Clipboard-2");

selectWindow("Result of Clipboard-1");

imageCalculator("Subtract create", "Result of Clipboard-1","Clipboard");

selectWindow("Result of Result of Clipboard-1");

run("Watershed");

run("Analyze Particles...", "size=10-Infinity circularity=0.00-1.00 show=Nothing add");

roiManager("Show All with labels");

roiManager("Show All");

roiManager("Combine");

run("Close");

run("Add to Manager");

selectWindow("Result of Clipboard-1");

close();

selectWindow("Clipboard-1");

close();

selectWindow("Clipboard");

close();

selectWindow("Clipboard-2");

close();

Macro3

setAutoThreshold();

setThreshold(400, 99999);

run("Convert to Mask");

run("Analyze Particles...", "size=10-1000 circularity=0.00-0.50 show=Masks clear record");

run("Add to Manager ");

Macro4

rename("Stack");

run("Select All");

run("Copy");

run("Internal Clipboard");

run("Subtract Background...", "rolling=200 slice");

setAutoThreshold("Default dark");

**Manual thresholding of the image**

run("Watershed");

run("Skeletonize");

roiManager("Select", 0);

run("Analyze Particles...", "size=10-Infinity add");

roiManager("Select", 0);

roiManager("Delete");

roiManager("Select All");

roiManager("Combine");

roiManager("Add");

selectWindow("Clipboard");

close();

selectWindow("Stack");

run("Z Project...", "start=2 projection=[Average Intensity]");

resetMinAndMax();

setMinAndMax(200, 1200);

run("8-bit");

run("Convolve...", "text1=[-1 -1 -1 -1 -1\n-1 -1 -1 -1 -1\n-1 -1 30 -1 -1\n-1 -1 -1 -1 -1\n-1 -1 -1 -1 -1\n] normalize");

setAutoThreshold("Default dark");

setThreshold(155, 255);

setOption("BlackBackground", true);

run("Convert to Mask");
