# Supplementary material for: MAP6 interacts with Tctex1 and Cav2.2/N‐type calcium channels to regulate calcium signalling in neurons
Source: Eur J Neurosci. 2017 Nov 22;46(11):2754–67. doi: 10.1111/ejn.13766 (PMC5765474; doi:10.1111/ejn.13766)
Supplement: Supplementary file 2 [file EJN-46-2754-s002.pdf]

EJN European Journal of Neuroscience FENS Federation of European Neuroscience Societies

MAP6 interacts with Tctex1 and Ca v2.2/N-type calcium channels to regulate calcium signaling in neurons

Jacques Brocard, Fabrice Dufour, Sylvie Gory-Fauré, Christophe Arnoult, Christophe Bosc, Eric Denarier, Leticia Peris, Yasmina Saoudi, Michel De Waard and Annie Andrieux

Review timeline:

Submission date:	05 July 2017
Editorial Decision:	18 August 2017
Revision received:	20 September 2017
Editorial Decision:	17 October 2017
Revision received:	20 October 2017
Accepted:	23 October 2017

Editor: Laurent Fagni

1st Editorial Decision 18 August 2017

Dear Dr. Andrieux,

Your manuscript was reviewed by three external reviewers as well as by the Section Editor, Dr. Laurent Fagni, and ourselves. The reviews collectively indicate that your experiments generated new and important information. However, there are several substantial issues that need to be clarified/resolved before we can consider your manuscript further for publication in EJN.

As indicated in his report, referee #1 was mainly concerned about discrepancies between results obtained from electrophysiological and Ca2+ imaging, as well as the role of Ca stores. As suggested by referee 2, you will need to justify the absence of effect of MAP6 over-expression in wild type neurons. The referee was also concerned about possible involvement of T-type and other types of Ca2+ channels. Both referees also mentioned a lack of explanation about pairing of data obtained from wild type and KO cells. All these points and other comments raised by the referees should be satisfied before we can further consider this paper.

Please also attend to the following issues:
1) We would greatly prefer if the results were not discussed in the introduction
2) More details are needed regarding your yeast screening procedures
3) You must provide a statement of ethical approval
4) Please make clear precisely the number of animals used.
5) Please make clear how animals were euthanized.
6) Your data sharing statement needs to be in its own section
7) Replace your barcharts with scatterplots or some other more informative hybrid depictions - see http://onlinelibrary.wiley.com/doi/10.1111/ejn.13400/epdf
8) Fig 5d: green and yellow text is really hard to read and needs to be changed.

If you are able to respond fully to the points raised, we would be pleased to receive a revision of your paper within 12 weeks.

Thank you for submitting your work to EJN.

Kind regards,

John Foxe & Paul Bolam
co-Editors in Chief, EJN

Reviews:

Reviewer: 1 (Ji-Fang Zhang, Thomas Jefferson University, USA)

Comments to the Author

EJN European Journal of Neuroscience FENS Federation of European Neuroscience Societies

MS No. EJN-2017-07-24763 --- "MAP6 interacts with Tctex1 and Cav2.2/N-type calcium channels to regulate calcium signaling in neurons"

In this manuscript, the authors reported their finding of the role of MAP6 in Cav channel trafficking. Starting with yeast two-hybrid screening, the authors identified Tctex1 as a partner protein for MAP6. This initially discovery was followed with a series of experiments to demonstrate the potential functional role of Tctex1-MAP6-CaV2.2 channel interactions. While I find the results interesting, there are issues which need clarified by the authors.

(1) Discrepancy between electrophysiology results (Fig 2A) and results of Ca2+ imaging and immunostaining. Fig2A shows a significant reduction (~25%) of the whole cell recording of Ca2+ currents between WT and MAP6 KO in the presence of nifedipine. Since the CaV1 channels are blocked and the whole cell current comes from the cell body, difference represents mostly reduction of CaV2 channels on the cell body. However, Ca2+ imaging (eg Fig 2) and immunostaining (Fig 4) did not seem to reflect such significant reduction of CaV2 Ca2+ channels. A simple interpretation is that the electrophysiology recording reflects the functional Ca2+ channels on the plasma membrane (cell body) while the other methods, as they were used in the manuscript, are not as sensitive or clean. For instance, neurons were permeabilized for immunostaining, thus, the results represent a mixed signal of CaV2.2 channels on the plasma membrane as well as inside the cell. Therefore, there could be a reduction of CaV2.2 channels on the plasma membrane of the cell body, but, the method itself is not appropriate to detect such differences (Fig 4A).

(2) While I like the approach of measuring the Ca2+ signal using Ca2+ imaging, it seems that the approach is overly simplified, particularly, the authors did not mention any potential complication by intracellular Ca2+ stores to their results. For instance, it is well known that Ca2+ can trigger release of Ca2+ from internal Ca2+ stores (Ca2+ induced Ca2+ release). Would it be possible that complications from internal Ca2+ stores might make the approach less sensitive to show the reduction of CaV2.2 on the cell membrane?

In Fig 3A, it is not clear to me how the authors paired the neurons from the WT culture to that of MAP6 KO. These are independent neuronal cultures from WT and KO mice. It is not like the before and after experiments, where the same neuron might exhibit different outcome. Also, it is not clear whether efforts were made to prevent non-specific "sticking" of the peptide toxins to the recording system, such as tubing, recording chamber, etc. Such non-specific sticking of the peptide to the recording system will effectively reduce the concentrations of the peptide toxins in the solution, resulting inconsistent results. Reagents, such as cytochrome C is often used to minimize such non-specific sticking.

In Fig 3C, the left panel shows the KO neuron has a high Ca2+ signal intensity that WT, in contrast to the right panel and the texts. Mislabeling?

(3) It is not clear to me what exactly the authors meant by "decreased clathrin-mediated recycling" in MAP6 KO neurons? Clathrin is known to be part of endocytosis machinery, involved in internalization of membrane proteins. Clathrin is not known for re-insertion of the membrane protein back to the plasma membrane. So, if the clathrin-mediated process is impaired in KO neurons, would it be that more CaV channels would remain on the plasma membrane?

(4) In the methods section, should a statement be made regarding the animal welfare in preparation of neuronal cultures?

Reviewer: 2 (Norbert Weiss, Academy of Sciences of the Czech Republic, Czech Republic)

Comments to the Author
In this study, Brocard et al., analyze the functional role of MAP6 in the control of voltage-gated calcium channel expression. Using MAP6 deficient hippocampal and cortical neurons in culture, they demonstrate that functional expression of Cav2.2 channels is altered in MAP6 KO cells, possibly due to a trafficking defect. In addition, they demonstrate the existence of a protein complex between MAP6/Tctex1 and Cav2.2 channels, and that functional expression of Cav2.2 in MAP6 KO neurons can be restored upon re-expression of MAP6.

The experiments are in general well performed and the manuscript is well written and contains some interesting findings but also weaknesses that confound the results and conclusions. For instance, it remains unclear how MAP6 modulates the functional expression of Cav2.2 channels. Below are some suggestions/comments that the authors might consider.

Major comments:

1- Figure 1E and E: please provide quantification for the co-immunoprecipitations of MAP6 mutants with

Tctex1.

2- According to representative barium current traces shown in Fig. 2A, not only the current density is decreased in MAP6 KO hippocampal neurons, but the current kinetics are also altered. Could the authors comment on this and/or provide additional analysis?

3- While re-expression of MAP6-E in MAP6 KO cells nicely restored KCL-induced intracellular calcium elevations to the level of the WT (Fig. 2E), it had no effect on WT neurons. If MAP6 contributes to the trafficking of Cav channels, one could have expected an increased calcium level in WT neurons overexpressing MAP6-E. The authors may comment on this.

4- In their calcium imaging experiments, the authors used KCL+nimodipine/KCL peak ratios to isolate the “net contribution of Cav2-type calcium channels to
increases of cytoplasmic calcium during KCl stimulations”. However, there is no T-type channel blocker in the recording solution and therefore the authors looked at the contribution of both Cav2 and Cav3 channels.

5- Figure 3A: considering that the data collected in WT cells are independent from those collected in MAP6 KO neurons, it is not clear how these data can be paired with each other. The authors should use a similar representation as in Fig. 2C-E and provide the corresponding statistical analysis.

6- Figure 3C: based on the observation that spontaneous calcium activity is decreased in neurites of hippocampal MAP6 KO cells, the authors state that “these results indicated that Cav2.1/PQ-type calcium channels may be diminished in mature MAP6 KO neuronal networks, thus mediating less intense spontaneous activity than in WT neurons”. There is no data to support this notion and alteration of other ion channels in MAP6 KO cells could have altered spontaneous electrical activity. The authors should at least present evidence that differential spontaneous calcium activity between WT and MAP6 KO cells is abolished in the presence of [illegible]-agatoxin IVA.

7- Figure 4B and C: the authors show a decreased total expression of Cav2.2 in neurites of MAP6 KO cells but not in the soma. In contrast, KCL-induced calcium elevations is observed both in the soma and neurites (Fig. 2C-D), and decreased functional expression of somatic Cav2 channels is further confirmed by electrophysiological recordings (that essentially asses somatic channels; Fig. 2A). Could the authors comment on this? A specific measurement of the expression of Cav2.2 at the cell surface instead of the total expression would be required to better understand whether MAP6 modulates expression of the channel protein in the plasma membrane or rather channel gating?

8- Figure 5A and B: Please provide the quantification for co-immunoprecipitation experiments.

9- Figure 5C: the rescue experiments using various MP6 mutants are normalized to the respective sham values obtained in WT and MAP6 KO cells. Although this representation allows for comparison of the rescue ability between MAP6 mutants, it would be useful to express data obtained in MAP6 KO as a function of the WT values to present the “real rescue” compared to WT conditions.

Minor comment/editing:

-page 25: In the Discussion, the authors indicate that “a minor decrease in surface expression of Cav2.1/PQ-type channels was uncovered in mature neural networks”. This statement should be removed of rephrased, as there is no supporting experimental data.

-page 26: In the Discussion, the authors justify the absence of effect of overexpressed MAP6 on Cav expression in WT neurons with the requirement of “functional Tctex1-MAP6 pairs”. However, additional mechanisms limiting the amount of functional Cav2 channels at the cell surface have also been proposed (Cao YQ et al., J Neurosci 2010; Hoppa MB et al., Nature 2012) and should be discussed.

Reviewer: 3 (Martin Heine, Leibniz Institute for Neurobiology, Germany)

Comments to the Author
The paper by Brocard et al. focus on the trafficking of calcium channels to the neuronal surface. The study focused on the MAP6 protein and the relation to calcium signalling. The work includes several experimental approaches to confirm the proposed dependence of Cav2-channel traffic on the presence of MAP6. The paper show new interactions between the Cav2.2-channel and MAP6. The interesting point is the triple complex between channel/MAP6 and Tctex1 that has been proposed to be a tuning element for the surface delivery of the calcium channels. Here I have a couple of questions:

The proposed local importance of the complex is evidenced by the unchanged labeling of channels in MAP6 KO but the loss of current density. Is there a possibility to show a relevant impact on presynaptic channel

abundance? The mentioned hypoglutamatergy in MAP6 KO and the proposed upregulation of NMDAR might be an indicator? Since neurons are very polarized cells and difficult to control by patch-clamp the local analysis of calcium signals/vesicular release in combination with presynaptic markers (for example uptake of fluorescent synaptotagmin antibodies) would be very helpful to demonstrate the impact of traffic mismatch under the KO-conditions. The data represented for spontaneous activity point in this direction but are difficult to interpret, since primary cultures can be very variable in their developmental stage between 10-24 DIV. A more focused analysis of local channel abundance would be very informative.

The overall turnover of the channels has been proposed to be in the range of 48h, might this be also biased by the rather global approach of calcium measurements in the soma and neurite.

Technical points:
For the WB analysis in figure 1F, it would be needed to show a clear example of MAP6-WB, since the example in the figure shows several bands sometimes merged together. Same is through for Fig5A, B.
In Fig2B, the example traces and bar graph for the neuritic calcium response should have a different color, since yellow on a with background is not convenient to read.

In Fig.4B, C examples are presented in green and red and merged, please use green and magenta to be more clear.

The sketch of the proposed function of MAP6 for the channel traffic is helpful but due to the color code difficult to read.

Within the text there are several spelling mistakes that need to be addressed.

Authors' Response 20 September 2017

Dear Editors,

Please find enclosed a resubmitted manuscript entitled **"MAP6 interacts with Tctex1 and Ca_v2.2/N-type calcium channels to regulate calcium signaling in neurons"** by Brocard *et al.* that we wish to publish in *European Journal of Neuroscience*.

We want to thank the reviewers and the editors for their comments about our manuscript. They have been taken into account to improve it as detailed below (new text was underlined in the manuscript as recommended).

Should you have any questions or comments on the suitability of this manuscript, please do not hesitate to contact us.

Sincerely yours,

Dr Annie Andrieux

Comments from the editors:
1) We would greatly prefer if the results were not discussed in the introduction
The last paragraph of the Introduction section was reduced to one sentence, accordingly.

2) More details are needed regarding your yeast screening procedures
The corresponding M&M paragraph was expanded and entirely rewritten.
3) You must provide a statement of ethical approval
Such a statement was added to an expanded "Animal research & breeding" paragraph of the M&M section.

4) Please make clear precisely the number of animals used.
The total number of pregnant females used for neuronal cultures have been indicated in the "Animal research & breeding" paragraph.

5) Please make clear how animals were euthanized.
A new sentence was added to the "Neuronal cultures" paragraph, clarifying how animals were euthanized.

6) Your data sharing statement needs to be in its own section
The data sharing statement has been moved to its own paragraph "Data Accessibility" (p27).

EJN European Journal of Neuroscience FENS Federation of European Neuroscience Societies

7) Replace your barcharts with scatterplots or some other more informative hybrid depictions - see http://onlinelibrary.wiley.com/doi/10.1111/ejn.13400/epdf
All the barcharts have been changed to scatterplots as required.

8) Fig 5d: green and yellow text is really hard to read and needs to be changed.
Colors of Fig. 5D were modified accordingly.

Comments from the Reviewer: 1
(1) Discrepancy between electrophysiology results (Fig 2A) and results of Ca2+ imaging and immunostaining.
As stated by the reviewer, the three techniques used (electrophysiological measurements, calcium imaging and immunolabeling) are different and yield contrasting results from which it is possible to draw conclusions. Whole cell electrophysiological measurements displayed the largest reduction of calcium currents through the plasma membrane of the cell bodies and proximal neurites of MAP6 KO neurons. Since these experiments were conducted in the presence of TTX (Nav channel inhibitor) and nifedipine (Cav1 channels inhibitor), they are probably the most specific but do not document the regional specificity of this reduction. Calcium imaging on the other hand is far less specific as Na+ influxes are not inhibited and may be followed by Na/Ca exchange - although this has been limited by using a Li-containing medium - in addition to Cav-mediated calcium influx. However, this technique enabled the segmentation of the cell bodies and the neurites from the observed neurons and led to the conclusion that neuritic decrease in calcium signaling was even higher than that observed in the cell bodies. Finally, immunostaining did not indicate a global decrease of Cav2.2 calcium channels, thus suggesting that the location of the channels, not their production and/or degradation, may be modified in the absence of MAP6.

(2) While I like the approach of measuring the Ca2+ signal using Ca2+ imaging, it seems that the approach is overly simplified.
"Concerning any potential complication by intracellular Ca2+ stores to [our] results", we note that calcium influxes elicited by depolarization are barely shaped by intracellular stores (Stanika *et al.*, 2012) (Friel, 2004). Moreover, the mitochondrial capacity to buffer calcium during KCl stimulations in MAP6 KO neurons seemed to be intact as compared to WT neurons in our preliminary experiments (see below). However, we cannot overrule the possible interplay between intracellular stores and spontaneous calcium activity as measured in Fig. 3C. The corresponding paragraph in the Discussion has been modified accordingly (p24-25).

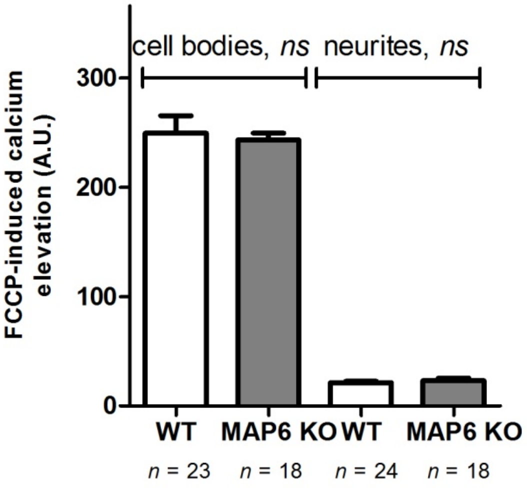

In Fig 3A...
Data pairing used in this figure is technical only. A full P24 plate of cortical neurons was treated with various toxins at a given time but measuring KCl-elicited calcium elevation throughout the plate needed 12 min (30 sec per well). To eliminate possible influence of the time delay on our measurements, we elected to pair the measurements obtained from MAP6 KO wells to the those obtained from their neighboring WT wells 30 sec later. Accurate description of the tests performed was removed from the "Statistical Analysis" paragraph of the M&M and added to the Fig. 3 caption instead.

Also, it is not clear whether efforts were made to prevent non-specific "sticking" of the peptide toxins to the recording system, such as tubing, recording chamber, etc.
Actually the toxins were never perfused but added directly from an intermediate tube where they were mixed with the proper medium. Moreover, the same intermediate tube was used for the MAP6 KO wells and their paired WT wells, thereby increasing the necessity to consider technical pairing for statistical analysis.

In Fig 3C...

There is not mislabeling in this figure: while global calcium rise seems higher in the MAP6 KO example, it's the rapid spontaneous calcium variations whose intensity was measured and reported on the right panel.

(3) It is not clear to me what exactly the authors meant by "decreased clathrin-mediated recycling" in MAP6 KO neurons?
The reviewer is correct in mentioning a false statement derived from our measurements of Tfn-containing vesicular contents of neuronal cultures: these vesicles are clathrin-coated at first but then lose the coat when they move back to the plasma membrane in a short recycling loop. The text has been modified accordingly (p21 and p25).

(4) In the methods section, should a statement be made regarding the animal welfare in preparation of neuronal cultures?
Such a statement was added at the beginning of the "Neuronal cultures" paragraph (p8).

Comments from the Reviewer: 2
1- Figure 1E and E: please provide quantification for the co-immunoprecipitations of MAP6 mutants with Tctex1.
The Western Blots of Fig. 1E and F have been quantified and figure caption was modified accordingly.

2- According to representative barium current traces shown in Fig. 2A, not only the current density is decreased in MAP6 KO hippocampal neurons, but the current kinetics are also altered. Could the authors comment on this and/or provide additional analysis?
We agree with the reviewer that the kinetics of the representative barium current traces as shown in Fig. 2A seem to be slowed in MAP6 KO neurons as compared to WT neurons. However, kinetics of currents in whole cell recordings depend on several technical factors, including the quality of the seal, the shape/size of the neuron body and the development of axonemal/dentritic tree in addition to the composition of the nanoenvironment of the calcium channels (Muller *et al.*, 2010). Accordingly, it did not seem reasonable to perform comparison of calcium current activations from the recordings displayed in Fig. 2A.

3- While re-expression of MAP6-E in MAP6 KO cells nicely restored KCL-induced intracellular calcium elevations to the level of the WT (Fig. 2E), it had no effect on WT neurons.
A full paragraph was actually devoted to this point just before conclusion (p26) and an alternate explanation for the absence of effect of overexpressed MAP6 on Cav expression in WT neurons was added in the Discussion section (p26) as requested by the reviewer (see below).

4- In their calcium imaging experiments, the authors used KCL+nimodipine/KCL peak ratios to isolate the “net contribution of Cav2-type calcium channels to increases of cytoplasmic calcium during KCl stimulations”. However, there is no T-type channel blocker in the recording solution and therefore the authors looked at the contribution of both Cav2 and Cav3 channels.
As the reviewer mentioned, we realized that our initial idea to measure "net contribution of Cav2-type calcium channels" was quite optimistic in view of the number of calcium transporters activated in neurons during depolarization. However, it is noteworthy that calcium signaling is mainly supported by Cav2.1 and Cav2.2 channels, with almost no participitation of Cav2.3 in cortical neurons (Schlick *et al.*, 2010) on the one hand and that T-type channel (Cav3-type channels) are saturated and thus inoperative during KCl-elicited depolarization (Huguenard, 1996) on the other hand.

5- Figure 3A: considering that the data collected in WT cells are independent from those collected in MAP6 KO neurons, it is not clear how these data can be paired with each other. The authors should use a similar representation as in Fig. 2C-E and provide the corresponding statistical analysis.
Data pairing used in this figure is technical only. A full P24 plate of cortical neurons was treated with various toxins at a given time but measuring KCl-elicited calcium elevation throughout the plate needed 12 min (30 sec per well). To eliminate possible influence of the time delay on our measurements, we elected to pair the measurements obtained from MAP6 KO wells to the those obtained from their neighboring WT wells 30 sec later. Accurate description of the tests performed was removed from the "Statistical Analysis" paragraph of the M&M and added to the Fig. 3 caption instead.

6- Figure 3C: There is no data to support this notion and alteration of other ion channels in MAP6 KO cells could have altered spontaneous electrical activity.

EJN European Journal of Neuroscience
FENS Federation of European Neuroscience Societies

Of course, the reviewer is correct to mention that there is no direct evidence that $Ca_v2.1$/PQ-type calcium channels are missing in MAP6 KO neurons. This is why we measured spontaneous activity in mature neuronal networks where it was shown that $Ca_v2.1$/PQ-type calcium channels are the main actor of calcium signaling (Qian & Noebels, 2001). Since the intensity of spontaneous calcium activity in MAP6 KO neurons was decreased as compared to WT, we assumed "that $Ca_v2.1$/PQ-type calcium channels may be diminished in mature MAP6 KO neuronal networks". Since we also agree that other ion channels may also be involved, we added an alternate explanation in the corresponding paragraph of the Discussion section (p24-25).

7- Figure 4B and C: A specific measurement of the expression of Cav2.2 at the cell surface instead of the total expression would be required to better understand whether MAP6 modulates expression of the channel protein in the plasma membrane or rather channel gating?
We agree with the reviewer that measuring Cav2.2 expression in the only plasma membrane would be the ideal readout for this study. Unfortunately, we did not succeed in performing such measurements with cell surface biotinylation although we did detect the plasma membrane content of the Na/K exchanger for instance (see opposite). This is the reason why we used different technical angles to show univocally the decrease of Cav2.2 channels-supported calcium influxes in MAP6 KO neurons.

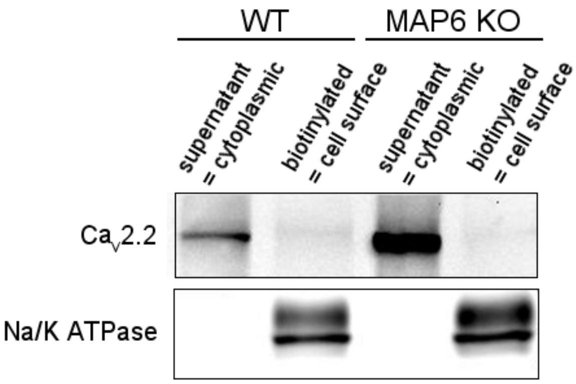

8- Figure 5A and B: Please provide the quantification for co-immunoprecipitation experiments.
The Western Blots indicated have been quantified and figure caption was modified accordingly.

9- Figure 5C: it would be useful to express data obtained in MAP6 KO as a function of the WT values to present the “real rescue” compared to WT conditions.
While there is merit to this kind of representation, it did not seem relevant to our attempt to demonstrate that overexpressing MAP6-E in MAP6 KO neurons may increase neuritic Cav2.2/N-type calcium channels. It may also lead to a significant loss of power as more Multiple Comparison Tests are performed within the framework of a global ANOVA comparison. However, we agree with the reviewer that comparing the overall restoration obtained in MAP6 KO neurons with the initial levels of neuritic Cav2.2/N-type calcium channels measured in WT neurons deserves a better representation. Accordingly, we introduced a new supplementary figure (Fig. S3) at the end of the last paragraph of the Results section (p23).

-page 25: In the Discussion, the authors indicate that “a minor decrease in surface expression of Cav2.1/PQ-type channels was uncovered in mature neural networks”. This statement should be removed of rephrased, as there is no supporting experimental data.
This sentence was removed entirely.

-page 26: In the Discussion, the authors justify the absence of effect of overexpressed MAP6 on Cav expression in WT neurons with the requirement of “functional Tctex1-MAP6 pairs”. However, additional mechanisms limiting the amount of functional Cav2 channels at the cell surface have also been proposed (Cao YQ et al., J Neurosci 2010; Hoppa MB et al., Nature 2012) and should be discussed.
An alternate explanation for the absence of effect of overexpressed MAP6 on Cav expression in WT neurons was added in the Discussion section (p26).

Comments from the Reviewer: 3
- Is there a possibility to show a relevant impact on presynaptic channel abundance?
While the authors agree with the reviewer that measurement of plasma membrane channel abundance would be an ideal readout for our experiments, we did not succeed in performing such measurements with cell surface biotinylation (see above, response to reviewer2).

- The mentioned hypoglutamatergy in MAP6 KO and the proposed upregulation of NMDAR might be an indicator?
Yes, as mentioned in the Discussion section, the observations made in the presence of kynurenic acid indicated that more NMDAR or more sensitive NMDAR were expressed in MAP6 KO neurons.

- The data represented for spontaneous activity point in this direction but are difficult to interpret, since primary cultures can be very variable in their developmental stage between 10-24 DIV. A more focused analysis of local channel abundance would be very informative.
We agree with the reviewer that "A more focused analysis of local channel abundance would be very informative" but we believe this could be the matter for a follow up study.

- The overall turnover of the channels has been proposed to be in the range of 48h, might this be also biased by the rather global approach of calcium measurements in the soma and neurite.
Yes, the overall turnover mentioned in the Discussion section was a reference to the initial study by Lai *et al*. (2005) where neurons inhibited for Tctex1's interactions with $Ca_v2.2$/N-type channels needed more than 48 hours to display a measurable decrease in electrophysiological recordings, i.e. in cell bodies and proximal neurites. It is in no case an estimate of the channel turnover in the presynaptic boutons and again, we believe this could be the matter for a follow up study.

- For the WB analysis in figure 1F, it would be needed to show a clear example of MAP6-WB
Unfortunately, the plasmids encoding the various MAP6-E mutants (from a pcDNA3.1 backbone) produce degradation bands in addition to the main expected band at the proper weight. These smeared signals do not correspond to non-specific detection as there is no signal when the immunoprecipitation is performed from extracts of sham-transfected cells. Moreover, this difficulty is specific to these constructs as we performed elsewhere immunoprecipitations of endogenous MAP6-E with no such problems [Fig. 7, (Deloulme *et al.*, 2015)]. A note summarizing this issue was added in the captions of Fig. 1F, 5A and 5B.

- In Fig2B, the example traces and bar graph for the neuritic calcium response should have a different color, since yellow on a with background is not convenient to read.
Accordingly the yellow color was modified to green.

- In Fig.4B, C examples are presented in green and red and merged, please use green and magenta to be more clear.
The changes have been made according to the reviewer's recommendations.

- The sketch of the proposed function of MAP6 for the channel traffic is helpful but due to the color code difficult to read.
Colors of Fig. 5D were modified accordingly.

- Within the text there are several spelling mistakes that need to be addressed.
Every text part has been painstakingly verified, in addition to using the Windows spell-checker, to remove any residual spelling mistake.

References

Deloulme, J.C., Gory-Faure, S., Mauconduit, F., Chauvet, S., Jonckheere, J., Boulan, B., Mire, E., Xue, J., Jany, M., Maucler, C., Deparis, A.A., Montigon, O., Daoust, A., Barbier, E.L., Bosc, C., Deglon, N., Brocard, J., Denarier, E., Le Brun, I., Pernet-Gallay, K., Vilgrain, I., Robinson, P.J., Lahrech, H., Mann, F. & Andrieux, A. (2015) Microtubule-associated protein 6 mediates neuronal connectivity through Semaphorin 3E-dependent signalling for axonal growth. *Nat Commun*, **6**, 7246.

Friel, D. (2004) Interplay between ER Ca2+ uptake and release fluxes in neurons and its impact on [Ca2+] dynamics *Biological Research*, **37**, 665-674.

Huguenard, J.R. (1996) Low-threshold calcium currents in central nervous system neurons. *Annu Rev Physiol*, **58**, 329-348.

Muller, C.S., Haupt, A., Bildl, W., Schindler, J., Knaus, H.G., Meissner, M., Rammner, B., Striessnig, J., Flockerzi, V., Fakler, B. & Schulte, U. (2010) Quantitative proteomics of the Cav2 channel nano-environments in the mammalian brain. *Proc Natl Acad Sci U S A*, **107**, 14950-14957.

Qian, J. & Noebels, J.L. (2001) Presynaptic Ca2+ channels and neurotransmitter release at the terminal of a mouse cortical neuron. *J Neurosci*, **21**, 3721-3728.

EJN European Journal of Neuroscience FENS Federation of European Neuroscience Societies

Schlick, B., Flucher, B.E. & Obermair, G.J. (2010) Voltage-activated calcium channel expression profiles in mouse brain and cultured hippocampal neurons. *Neuroscience*, **167**, 786-798.

Stanika, R.I., Villanueva, I., Kazanina, G., Andrews, S.B. & Pivovarova, N.B. (2012) Comparative impact of voltage-gated calcium channels and NMDA receptors on mitochondria-mediated neuronal injury. *J Neurosci*, **32**, 6642-6650.

2nd Editorial Decision 17 October 2017

Dear Dr. Andrieux,

Your revised manuscript was re-evaluated by external reviewers as well as by the Section Editor, Dr. Laurent Fagni and ourselves. We are pleased to inform you that it will be accepted for publication in EJN after you have dealt with a couple of minor points.

Please address the queries raised by Reviewer 1 concerning the statistical analyses and please also provide a graphical abstract.

If you are able to respond fully to the points raised, we shall be pleased to receive a revision of your paper within 30 days.

Thank you for submitting your work to EJN.

Kind regards,

Paul Bolam & John Foxe
co-Editors in Chief, EJN

Reviews:

Reviewer: 1 (Ji-Fang Zhang, Thomas Jefferson University, USA)

Comments to the Author
MS No. EJN-2017-07-24763.R1 --- "MAP6 interacts with Tctex1 and Cav2.2/N-type calcium channels to regulate calcium signaling in neurons"

In this revised manuscript, the authors addressed most of the issues raised by reviewers. No new data were provided. There are still two issues:

(1) Again, pairing in Fig. 3A: From the authors' explanation, I understand better the reason for such pairing in that figure, basically trying to minimize potential time-dependent variation of the imaging data. However, once the data were paired, a different statistical method, Wilcoxon test, was used. I am not a statistician, but, by definition, A Wilcoxon test is a nonparametric test that can be used to determine whether two dependent samples are selected from populations having the same distribution. It can be used as an alternative to the paired Student's t-test or t-test for matched pairs. As pointed out by both reviewer 1 and reviewer 2, WT neurons and MAP6 KO neurons are independent. It is not clear to me whether statistical tests, meant for paired samples, can be used in set of data.

A related issue: The authors stated "Note that Wilcoxon t-tests have been performed to account for technical data pairing between neighboring MAP6 KO and WT culture wells and this pairing was deemed as highly significant ($p < 0.01$) for each drug." ---- It is not clear to me what drugs were used for this set of data. I would have agreed that the Wilcoxon test would be appropriate for comparison of data before and after drug application.

(2) There is no indication whether the data presented in the MS are "mean +/- standard deviation" or "mean +/- standard error".

Reviewer: 2 (Norbert Weiss, Academy of Sciences of the Czech Republic, Czech Republic)

Comments to the Author
In this revised manuscript, the authors have properly answered most of the initial comments and the manuscript has been significantly improved. I have no additional comment and as far as I am concerned the manuscript can be considered for publication.

Authors' Response 20 October 2017

Authors' Response to Reviewers:
(1) Again, pairing in Fig. 3A: From the authors' explanation, I understand better the reason for such pairing in that figure, basically trying to minimize potential time-dependent variation of the imaging data.
>The reviewer is absolutely right and that is all there is to understand: we paired wells for technical reasons in order to minimize the variations due to uncontrolled factors.

However, once the data were paired, a different statistical method, Wilcoxon test, was used. I am not a statistician, but, by definition, A Wilcoxon test is a nonparametric test that can be used to determine whether two dependent samples are selected from populations having the same distribution.
>Although a paired test may be used to determine whether two dependent samples are selected from populations having the same distribution, it can ALSO be used as an alternative to a Student's t-test when data distribution is not gaussian. From Wikipedia:
"The Wilcoxon signed-rank test is a non-parametric statistical hypothesis test used when comparing two related samples, matched samples, or repeated measurements on a single sample to assess whether their population mean ranks differ (i.e. it is a paired difference test). It can be used as an alternative to the paired Student's t-test, t-test for matched pairs, or the t-test for dependent samples when the population cannot be assumed to be normally distributed.
[1] A Wilcoxon signedrank
test is a nonparametric test that can be used to determine whether two dependent samples were selected from populations having the same distribution."

It can be used as an alternative to the paired Student's t-test or t-test for matched pairs. As pointed out by both reviewer 1 and reviewer 2, WT neurons and MAP6 KO neurons are independent. It is not clear to me whether statistical tests, meant for paired samples, can be used in set of data.
>Although WT and MAP6 KO neurons are indeed independent, their well positions are not: the ones measured first give closer results to one another than the ones measured last. Hence the technical pairing.

A related issue: The authors stated "Note that Wilcoxon t-tests have been performed to account for technical data pairing between neighboring MAP6 KO and WT culture wells and this pairing was deemed as highly significant ($p < 0.01$) for each drug." ---- It is not clear to me what drugs were used for this set of data. I would have agreed that the Wilcoxon test would be appropriate for comparison of data before and after drug application.
>As mentioned in Fig. 3A's caption " Right panels, ratios of KCl-elicited fluorescence intensity peaks, recorded from WT (white squares) and MAP6 KO (grey squares) cortical neurons in the presence or absence of 20 µM nimodipine (yellow curves), 180 nM ω-agatoxin IVA (red curves) or 320 nM ω-conotoxin GVIA (green curves)." To add clarity to the figures themselves, the identity of the toxin used was added to the figure panels, directly. Furthermore, the decision leading to the pairing itself can be discussed based on its very testing, as provided by GraphPad. On the table below, the Wilcoxon signed rank test used for conotoxin-treated cultures (Fig. 3B, last panel in green) is deemed non-significant ($p=0.5830$) whereas the pairing itself, the fact that we decided to pair each well of MAP6 KO culture with its neighboring well of WT culture, IS highly significant ($p=0.0065$).

EJN European Journal of Neuroscience
FENS Federation of European Neuroscience Societies

t test	
Table Analyzed	drogues
Column D	WT
vs	vs
Column E	MAP6 KO
Wilcoxon signed rank test	
P value	0.5830
Exact or approximate P value?	Exact
P value summary	ns
Are medians signif. different? (P < 0.05)	No
One- or two-tailed P value?	Two-tailed
Sum of positive, negative ranks	62.00 , -43.00
Sum of signed ranks (W)	19.00
How effective was the pairing?	
rs (Spearman, Approximation)	0.6440
P Value (one tailed)	0.0065
P value summary	**
Was the pairing significantly effective?	Yes

(2) There is no indication whether the data presented in the MS are "mean +/- standard deviation" or "mean +/- standard error".
>Indication that data are "mean +/- standard error" was added at the end of the "Statistics" paragraph of the M&M section.
